# The structure of the monobactam-producing thioesterase domain of SulM forms a unique complex with the upstream carrier protein domain

**DOI:** 10.1016/j.jbc.2024.107489

**Published:** 2024-06-20

**Authors:** Ketan D. Patel, Ryan A. Oliver, Michael S. Lichstrahl, Rongfeng Li, Craig A. Townsend, Andrew M. Gulick

**Affiliations:** 1 Department of Structural Biology, University at Buffalo, SUNY, Buffalo, New York, USA; 2 Department of Chemistry, Johns Hopkins University, Baltimore, Maryland, USA

**Keywords:** nonribosomal peptide synthetase, NRPS, thioesterase, β-lactam antibiotic, antibiotics, monobactam, natural product biosynthesis, protein structure/function

## Abstract

Nonribosomal peptide synthetases (NRPSs) are responsible for the production of important biologically active peptides. The large, multidomain NRPSs operate through an assembly line strategy in which the growing peptide is tethered to carrier domains that deliver the intermediates to neighboring catalytic domains. While most NRPS domains catalyze standard chemistry of amino acid activation, peptide bond formation, and product release, some canonical NRPS catalytic domains promote unexpected chemistry. The paradigm monobactam antibiotic sulfazecin is produced through the activity of a terminal thioesterase domain of SulM, which catalyzes an unusual β-lactam–forming reaction in which the nitrogen of the C-terminal *N*-sulfo-2,3-diaminopropionate residue attacks its thioester tether to release the monobactam product. We have determined the structure of the thioesterase domain as both a free-standing domain and a didomain complex with the upstream *holo* peptidyl-carrier domain. The position of variant lid helices results in an active site pocket that is quite constrained, a feature that is likely necessary to orient the substrate properly for β-lactam formation. Modeling of a sulfazecin tripeptide into the active site identifies a plausible binding mode identifying potential interactions for the sulfamate and the peptide backbone with Arg2849 and Asn2819, respectively. The overall structure is similar to the β-lactone–forming thioesterase domain that is responsible for similar ring closure in the production of obafluorin. We further use these insights to enable bioinformatic analysis to identify additional, uncharacterized β-lactam–forming biosynthetic gene clusters by genome mining.

Many microbes produce a variety of natural products that are secreted from the cell where they play roles in adaptation to numerous changing environments. These small molecules may facilitate competition with other organisms or play a role in the acquisition of important nutrients. An important class of natural products are peptide and peptide-like molecules produced by the nonribosomal peptide synthetases (NRPSs). Not constrained by the synthetic limitations of the ribosome and amino acyl-tRNA synthetases, the NRPSs form products from hundreds of unique building blocks (1, 2). The diversity of the peptide products is enhanced not only by the wide spectrum of substrates but also by additional changes to cross-link, cyclize, and otherwise modify these molecules during their biosynthesis. NRPS products range in size from small cyclic dipeptides to large macrocycles containing more than twenty amino acids. Much effort has gone into the identification of NRPS biosynthetic gene clusters and the molecules that are produced by the encoded proteins, with multiple informatic tools available for their identification, analysis, and classification (3, 4, 5).

The NRPSs are large, modular proteins that function as assembly lines for the stepwise construction and extension of a peptide product. Each module is composed of a defined set of catalytic domains that are generally responsible for the incorporation of a single amino acid building block. Each module houses a peptidyl carrier-protein domain (PCP) that contains a conserved serine residue that is posttranslationally modified with a molecule of phosphopantetheine. The pantetheine thiol serves as the binding site for the activated amino acid substrate and the growing peptide. In addition to this carrier domain, NRPS modules generally contain adenylation domains that recognize and load the substrate onto the PCP and condensation domains that catalyze peptide bond formation simultaneously transferring the amino acid or upstream peptide to an amino acid that has been installed on a downstream PCP domain, thereby extending the peptide by one residue. While the covalent tethering of the nascent peptide to the PCP facilitates coordination of the catalytic steps, this feature also requires a final catalytic step to release the peptide from the terminal PCP domain. This step is most commonly carried out by a thioesterase domain that catalyzes hydrolysis of the peptide thioester with the pantetheine cofactor or, often, a macrocyclization step carried out when a nucleophilic group on the peptide attacks the thioester (6, 7). Cyclization can occur through the N-terminal amine of the peptide or internal side chain groups from lysine, serine, threonine, or other nucleophiles.

The thioesterase domain belongs to a large α/β hydrolase superfamily that catalyzes the hydrolysis of multiple substrates (8, 9). These enzymes contain a conserved fold of a central 7- or 8-stranded β-sheet with surrounding α-helices. Between strands β6 and β7 is a long loop generally containing two helices that form a lid over the active site. The catalytic machinery of the thioesterase domain contains a nucleophilic serine or less common cysteine residue that is activated by an aspartate and histidine dyad that form a hydrogen bonding network with the nucleophile (6, 9). The reaction mechanism employs two steps to release the peptide from the terminal PCP domain. First, the serine or cysteine attacks the thioester to transfer the peptide from the PCP and form an acyl-enzyme intermediate. This intermediate is resolved through a second off-loading step that is specific to the cyclization or hydrolysis necessary for each enzyme.

Previously, crystal structures have been determined for eight NRPS thioesterase domains. Unlike other NRPS catalytic domains, for which numerous structures have been solved complexed with informative ligands or paired with the carrier protein domains (10, 11, 12), the structural foundation of thioesterase domain function is much less well-characterized. There are only two structures of thioesterase domains that provide insight into their interactions with substrates. The thioesterase domains from the nocardicin and valinomycin pathways have been crystallized with ligands bound to the nucleophile to provide insight into the transiently formed acyl-enzyme intermediate. In the case of the thioesterase domain from the nocardicin protein NocB, a phosphonate analog of the peptide substrate was designed that reacted with the catalytic serine to identify the location of the terminal three residues of the peptide prior to release (13). In the valinomycin-producing Vlm2 enzyme, a protein engineering approach allowed the replacement of the catalytic residue with 2,3 diaminopropionate that reacted stably with the peptide (14). Finally, only a single thioesterase domain, present in the enterobactin NRPS EntF (15, 16), has been characterized as a complex with the PCP domain.

Among the most interesting and important NRPS products are peptide antibiotics that range in size as large as the glycopeptide and lipopeptide antibiotics containing ten or more amino acids (17, 18). Among the antibiotics produced by NRPS pathways are β-lactam antibiotics, such as penicillin, cephalosporin, and the monocyclic nocardicin and sulfazecin (19). Interestingly, the β-lactam ring is produced in these different molecules through different enzyme activities. A non-heme iron-dependent oxygenase catalyzes the production of the penicillins and cephalosporins from an NRPS tripeptide precursor that has been released from the enzyme (20). Other systems integrate the β-lactam–producing step directly into the NRPS assembly line, with ring formation occurring in the nocardicin pathway from an integrated condensation domain (21), while the activity of a thioesterase domain catalyzes the cyclization in sulfazecin biosynthesis (22, 23). These strategies in NRPS systems contrast with an ATP-dependent β-lactam synthetases that catalyze the ring closure in the production of the β-lactamase inhibitor clavulanic acid (24) and the broad-spectrum carbapenems (25, 26) by NRPS-independent pathways (27, 28).

Sulfazecin and its stereoisomer isosulfazecin were first identified in the early 1980s from a producer strain of *Pseudomonas acidophila* (29, 30). Sulfazecin consists of a tripeptide derivative formed from γ-glutamate, D-alanine, and an azetidin-2-one ring containing a sulfonate linkage to the nitrogen and a methoxy group added to the lactam ring. Sulfazecin binds to the *Escherichia coli* penicillin-binding proteins and is bacteriostatic at 6.25 μg ml^−1^. Isosulfazecin, the L-stereoisomer at the alanine residue, was reported to have slightly weaker antibacterial activity against some strains (31).

The biosynthetic gene cluster for sulfazecin was identified through transposon mutagenesis and screening for the inability to inhibit *E. coli* growth in a sensitive test strain. A single mutant strain was isolated from the producer *P. acidophila*, allowing the discovery of the biosynthetic cluster (22). We note that the producing organism has recently been reclassified and is recognized in NCBI as *Paraburkholderia acidicola* (32, 33).

The biosynthesis of sulfazecin (Fig. 1) employs a biosynthetic gene cluster containing two NRPS proteins. SulI (1089 residues) contains the initiation module that incorporates the γ-glutamate residue, while SulM (2984 residues) contains the second and third module (22). Also found within the cluster are SulG and SulH, involved in the production of L-2,3-diaminopropionate (L-DAP), and the sulfotransferase, SulN (23). The presence of SulG and SulH suggested that the β-lactam ring in sulfazecin might be derived from the cyclization of L-DAP. Biochemical analysis confirmed the incorporation of L-Ala and L-DAP by the adenylation domains of the SulM modules. A series of stepwise reconstitution experiments demonstrated that the NRPS assembly of the γ-D-Glu-D-Ala-L-Dap PCP3-bound tripeptide is *N*-sulfonated *in trans* by SulN and PAPS, which is then translocated (transacylation) to the SulTE active site Cys (23). Next, intramolecular 4-*exo*-trig cyclization is catalyzed to release the fully elaborated monobactam. This unprecedented process established the last known mechanism of β-lactam antibiotic biosynthesis (19, 23). Following release of the desmethoxy sulfazecin, the methoxy group is installed in two steps by the SulO and SulP enzymes.

**Figure 1:**
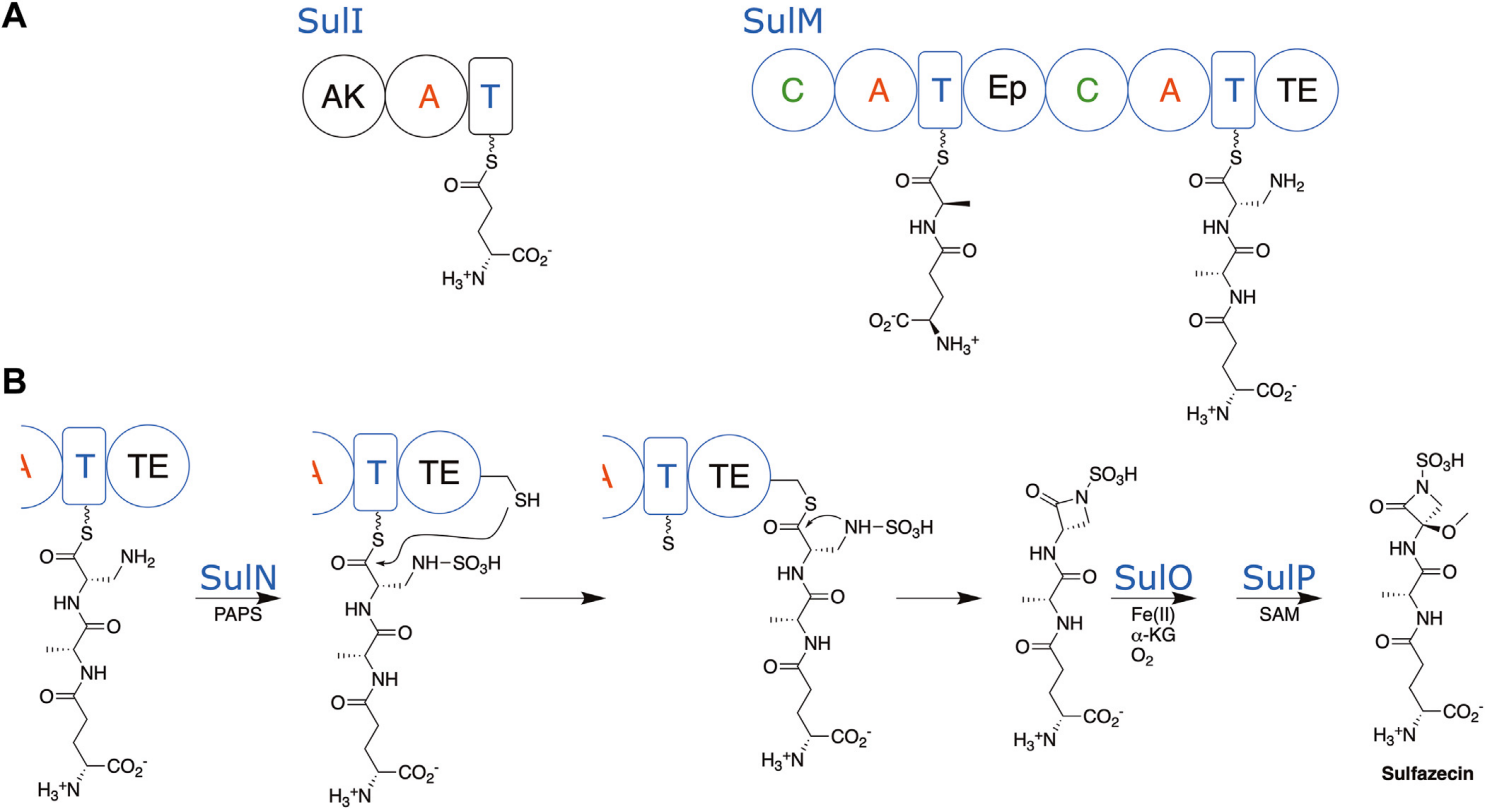
**Biosynthesis of sulfazecin.***A*, two NRPS proteins, SulI and SulM, harbor three modules necessary to produce the desmethoxysulfazecin. The SulI module contains an adenylylsulfate kinase, responsible for the production of PAPS, as well as the initiation module for D-glutamate. SulM contains two modules that incorporate L-alanine, which is epimerized to the D-stereoisomer in module 2, and L-DAP in module 3. *B*, the final steps in sulfazecin production include the *in trans* sulfonation catalyzed by SulN and then β-lactam formation by the thioesterase domain, resulting in the production of desmethoxysulfazecin. Two final steps catalyzed by the nonheme iron oxygenase SulO and methyltransferase SulP produce sulfazecin. NRPS, nonribosomal peptide synthetase.

The terminal β-lactam–forming catalytic activity of SulM TE domain motivated us to solve the structure of this domain. Here, we present the structure of the thioesterase domain on its own and as a complex with the upstream *holo*-PCP domain. The structure provides a view of the functional interaction between the carrier and the thioesterase domain, which illustrates a distinct interaction compared to EntF, the only prior structure of a PCP-thioesterase interaction. The structures are analyzed with molecular dynamics simulations, highlighting a stable conformation of the PCP–TE complex. Analysis of the active sites suggests that the thioesterase domains of β-lactam and β-lactone–forming enzymes provide a constrained architecture for the nucleophilic Cβ amine or hydroxyl of the C-terminal amino acid of the tripeptide to attack the thioester linkage for formation of the four-membered ring product.

## Results and discussion

### Structure of the SulM TE domain

There are currently structures for eight thioesterase domains (12) from modular NRPSs solved as single domain recombinant proteins or as part of a larger multidomain construct. The proteins represent distantly related enzymes, with amino acid pairwise sequence identities that range from 17 to 28% between different members (Fig. S1). The structures include the single domains from surfactin (34), fengycin (35), valinomycin (14), nocardicin (13), and skyllamycin (36) NRPS termination modules. Additionally, there are several structures of the thioesterase domains within larger multidomain protein structures including a PCP–TE complex from EntF (15, 16), which shows the interaction of the PCP and TE domains. The other NRPS thioesterase domains, involved in the production of surfactin (37), enterobactin (38), and obafluorin (39), as well as an uncharacterized NRPS from *Acinetobacter baumannii* (40), illustrate the position of the thioesterase domain relative to a complete termination module. In these structures, the thioesterase domain is highly dynamic and adopts multiple positions relative to the core of the module and does not interact functionally with the upstream PCP domains.

**Figure 2:**
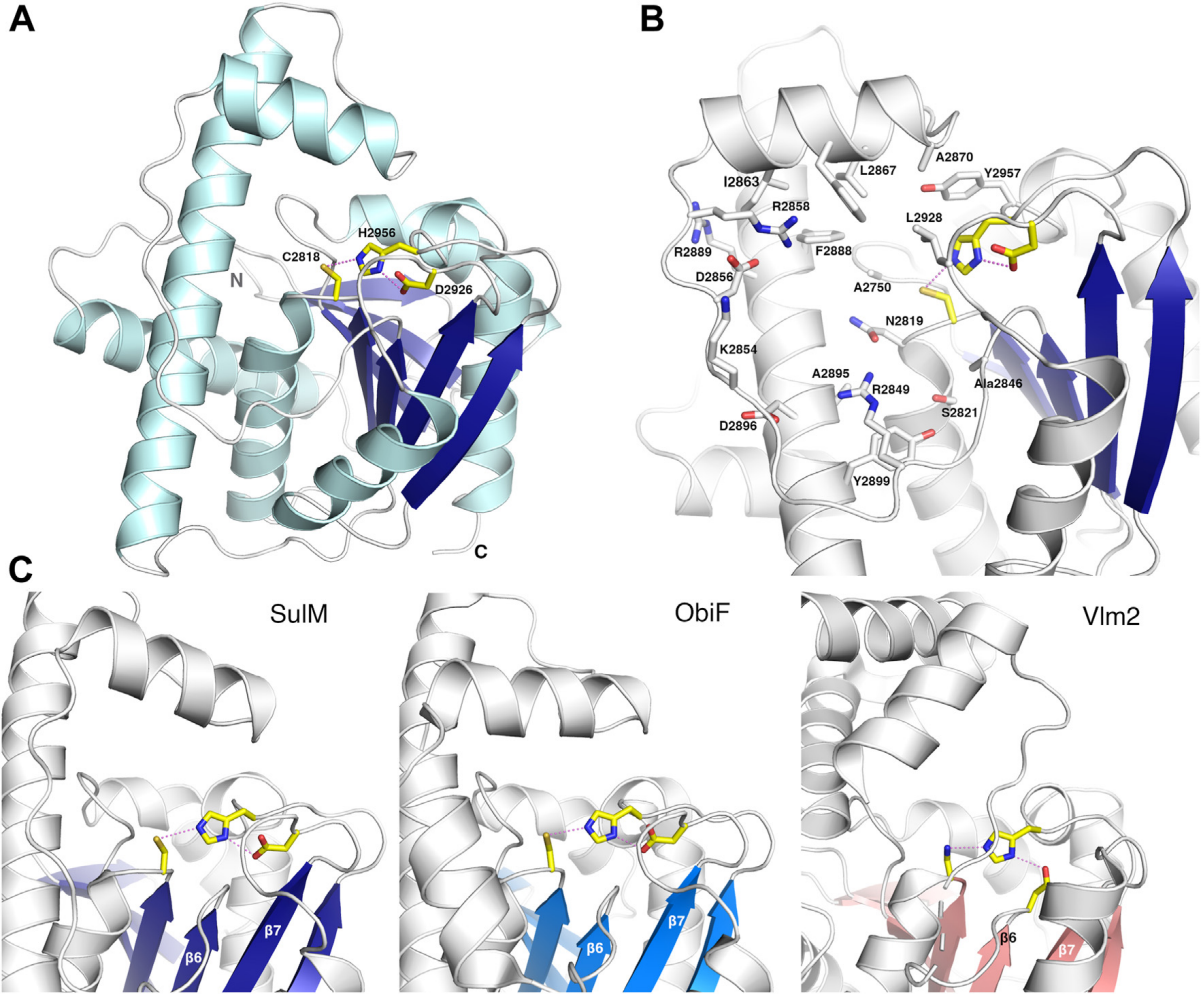
**Structure of SulM thioesterase domain.** The SulM thioesterase domain adopts a conventional α/β hydrolase fold with a central β-sheet surrounded on both sides by α-helices. *A*, the catalytic triad illustrates a nucleophilic cysteine residue Cys2818 that is positioned on the loop following strand β5, interacting with His2956 and Asp2926. *B*, the active site pocket is formed from residues on the lid loops and lid helices. *C*, SulM and ObiF thioesterase domains contain a catalytic triad in which the aspartic acid is positioned on the loop following strand β7. In contrast, the aspartic acid residue of Vlm2, like nearly all other structurally characterized NRPS thioesterase domains, is positioned in the loop following strand β6. NRPS, nonribosomal peptide synthetase.

To explore the structural foundation for β-lactam formation by the TE domain of SulM in sulfazecin biosynthesis, we determined the structure of the domain in the presence and absence of the partner carrier protein. Protein constructs from five cut sites upstream of the TE-coding sequence were selected for expression tests. The structure of the optimized genetically truncated thioesterase domain, which we refer to as SulTE, was determined (Fig. 2). The protein has a typical α/β hydrolase fold with seven central β strands (β2 to β8) flanked by four α helices on one side and three helices on another side (Fig. S2*A*). The first strand of the canonical α/β hydrolase fold is missing and hence the structure starts with the second β-strand, which is anti-parallel to the remaining six strands. A very long sixth α-helix along with seventh helix and the following loop (residues 2860–2877) form the lid region.

A comparison of the SulM thioesterase domain with earlier NRPS crystal structures shows that the protein adopts the conventional overall hydrolase fold. The root mean square (rms) displacement of pairwise comparisons of SulM with the remaining proteins ranges from 1.3 to 3.2 Å (Fig. S1), excluding the dynamic lid loops, with most pairwise comparisons showing rms displacements less than 2 Å.

The active site residues of the SulM thioesterase domain, Cys2818, Asp2926, and His2956, form the catalytic triad where the central histidine is 3.2 Å and 2.7 Å from the cysteine and aspartic acid residues, respectively. The nucleophile Cys2818 is present at the turn following the β5 strand, while Asp2926 is located on the loop that follows the β7 strand and His2956 is positioned on the loop after β8 strand.

Prior work (23) assessing the catalytic behavior of the SulTE domain proved far more difficult than our earlier experience with the dual function epimerase/hydrolase in nocardicin biosynthesis mediated by NocTE (21, 41). NocTE, like many thioesterase domains, could be evaluated by examining the fate of *N-*acetylcysteamine (SNAC) thioesters of potential peptide substrates. In contrast, examination of SulTE required a full reconstitution assay in which the pre-epimerized γ-D-Glu–D-Ala dipeptide was presented on PCP2 to recombinant SulM module 3 in the presence of L-Dap, ATP, SulN, and PAPS. The synthetic behavior of the SulTE active site variants C2818S showed rapid hydrolysis of the monobactam product generated *in situ*. On the other hand, the C2818A control showed no β-lactam formation and only very slow hydrolysis of the *N*-sulfonated PCP3-bound tripeptide thioester (23), similar to the active site serine to alanine variant of NocTE (41). In a further test of the SulTE catalytic triad, the D2926A variant was generated and assayed in a single but complete trial and found to be unreactive in both monobactam formation and hydrolysis. We attributed the absence of either reaction to reflect no substrate transfer from PCP3 to the TE active site.

The organization of the catalytic triad in SulTE is distinct from most NRPS thioesterase domains. Unlike the position of the aspartic acid residue on β6 (position I) seen in other NRPS TE domains, only ObiF1 (39) and SulTE reposition the aspartic acid residue to the loop following strand β7 referred as position II (Fig. 2*C*). This arrangement has also been observed in some type II proof-reading thioesterases such as RifR and RedJ (42, 43) and, indeed, is the more common configuration in other members of the α/β hydrolase superfamily (8, 9). To explore the impact of this altered positioning, the aspartic acid residue of ObiF1 was mutated to an alanine, resulting in a nearly 10-fold reduction in production formation (39). In ObiF, relocation of the aspartic acid to the location following strand β6 with a double mutant failed to restore activity, leading to the hypothesis that the unusual triad orientation seen in ObiF and SulM might enable an unusual positioning of the peptide for ring closure.

We compared the nature of the specific reaction chemistry (hydrolysis, cyclization, or β-lactam/lactone formation), the nature of the nucleophile (serine or cysteine), as well as the peptide substrate for the different NRPS enzymes to correlate with the unusual catalytic triad of the sulfazecin and obafluorin proteins. The thioesterase domains of both ObiF and SulM contain a cysteine as the catalytic nucleophile and both require the attack of the β-carbon hydroxyl or sulfamatae on the thioester to form the lactone or lactam ring, respectively. Thus, the relocation of the catalytic aspartic acid may facilitate the necessary configuration of the terminal residue of the peptide when bound as an acyl-enzyme intermediate to the nucleophilic cysteine to properly allow the monobactam or β-lactone–forming reaction to proceed. Of the other structurally characterized proteins, only the uncharacterized AB3403 from *A. baumannii* contains a nucleophilic cysteine, although it contains an aspartic acid residue of the triad that follows the β6 like other NRPS proteins (position I).

The other feature that stands out among the characterized NRPS systems is the size of the peptide product that will be situated on the catalytic nucleophile as part of the catalytic mechanism. The ultimate structures of the products released from these thioesterase domains (Fig. S3) highlight that the sulfazecin and obafluorin molecules are much smaller (mw < 370 Da) than the products of the other NRPS systems (mw ranging from 670 to 1470 Da). Thus, it is possible that the positioning of the catalytic triad in these NRPS enzymes with larger peptide products may be necessary to provide sufficient space in the active site to accommodate the large peptide.

### The lid helices of SulTE highlight structural diversity among the NRPS thioesterase domains

Alignment of the structurally characterized NRPS thioesterase domains illustrates that, despite the low sequence homology, the main secondary structural elements of the α/β superfamily superimpose for all family members. The distinguishing feature, however, in comparison of the structures is the region known as the lid loop. Thioesterase domains of the α/β superfamily generally contain two helices located between strands β6 and β7 (6). These two helices are positioned near the active site and interact with the loaded substrate in the acyl-enzyme complexes of NocB or Vlm2 (13, 14). We examined the NRPS thioesterase domain structures and noted remarkable diversity in the length and helical content of this lid region (Fig. S2*B*). The lid loop joining β6 and β7 ranges in size from 54 to 101 residues, containing as few as two and as many as five α-helices (Table S1).

The SulM thioesterase domain lid loop, which spans residues Gly2844 to Val2918, contains two helices. The second of these is the longest helix of a characterized NRPS thioesterase domain. This helix in the SulM thioesterase aligns with the final helix of all NRPS thioesterase structures. The SulM lid contains an extension of one additional turn of the helix compared to closest structural homolog ObiF and is longer by two to four helical turns than the other structures. The N-terminus of the helices align; however, they differ in the length with longer helices projecting away from the active site.

A second loop, joining strands β7 and β8, also adopts very different configurations in the different structures although it does not interact with ligands in the NocB or Vlm2 thioesterase domain structures (Fig. S2). While not as dramatic as the lid region, the loop joining β7 and β8 ranges from 19 to 26 residues in length, generally containing a single α-helix of 5 to 16 residues (Table S1). This second, variable loop contains no α-helices in the SrfA-C structure (34, 37), while the same region in the fengycin thioesterase domain (35) contains a split helix that is interrupted by a residue that adopts a left-handed helical conformation.

In the SulTE structure, the loop that follows the β7 strand runs from Gly2923 to Val2946 and contains an unusually long α-3_10_ composite helix. The first two residues of this helix, Tyr2932 and Pro2933, adopt a traditional α-helical conformation while the following nine residues form a 3_10_ helix. Among known NRPS thioesterase domain structures, only SulM and ObiF1 show α-3_10_ composite helix after β7 strand. As this loop contains the catalytic aspartic acid residue in these two proteins, the configuration of this loop may relate to the need to position this residue closer to the active site. In contrast, the loop after the β7 strand is further away from the active site in enzymes having the acidic aspartate residue after β6 strand (position I).

### Structure of the SulM TE domain in complex with the upstream PCP

**Figure 3:**
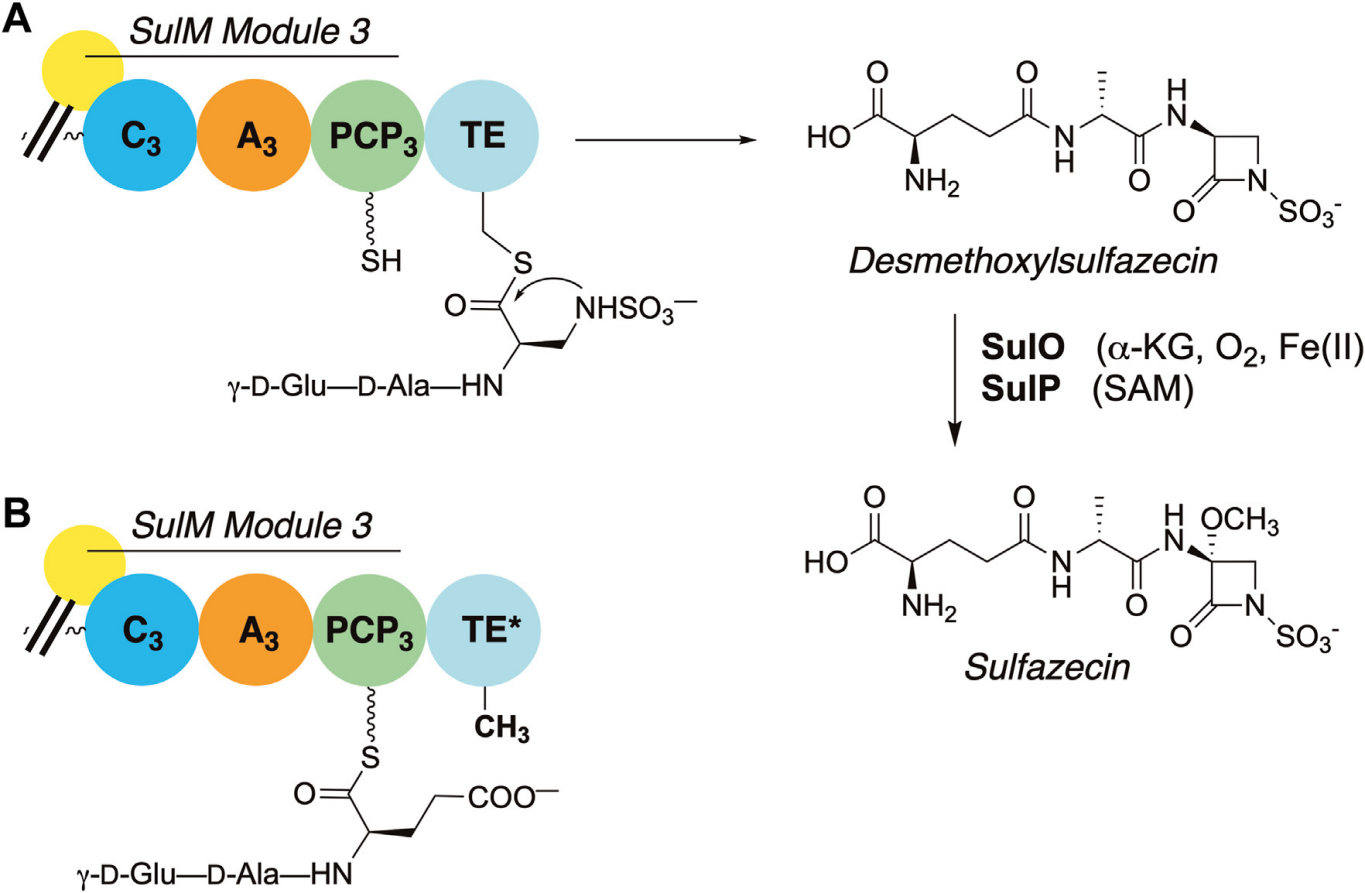
**Examination of monobactam formation in SulTE.***A*, γ-D-Glu-D-Ala-L-Dap linked to the pantetheine arm of PCP_3_ in the NRPS SulM is sulfonated *in trans* to the corresponding sulfamate and delivered to the SulTE catalytic Cys2818 by transthioesterification and cyclized to the monocyclic β-lactam product and released. Subsequent oxidation by SulO and *O*-methylation by SulP yield the monobactam sulfazecin. *B*, the *C*-terminal amino acid of the tripeptide in (*A*) was replaced with L-Glu to mimic the distal charge of the native sulfamate. NRPS, nonribosomal peptide synthetase.

We determined the didomain PCP-TE of SulM, in which the PCP was in the *holo* state loaded with the pantetheine cofactor. We designed a peptide mimic of the sulfazecin tripeptide by loading the pantetheine with the γ-D-Glu-D-Ala-L-Glu, where the C-terminal glutamate potentially forms anionic interactions of the natural *N-*sulfonated DAP substrate (Fig. 3). Additionally, the protein construct was derived from the C2818A mutant, which will be unable to initiate catalysis, with a goal of capturing the functional PCP–TE complex. This substrate surrogate was easily prepared analogously to the native γ-D-Glu-D-Ala-L-Dap sulfamate (23). The PCP-TE structure was determined and refined to a resolution of 2.73 Å resolution. The structure contained two independent chains in the asymmetric unit, each containing residues Glu2660 through Cys2979. The N-terminus of the protein also contained four residues from the purification tag, while the C-terminal five residues were disordered. The two chains superimposed with a rms displacement of 0.4 Å over the full length of both domains, indicating that the orientation of the PCP relative to the thioesterase domain was the same in both chains. Contiguous electron density the pantetheine was observed on Ser2688 of the PCP domains in both chains (Fig. S4).

The conserved position of the pantetheine attachment at the start of helix α2 of the PCP directs one face of the carrier domain towards the neighboring catalytic domains, with residues from α2, α3, and the loop joining helix α1 and α2 playing primary roles in the interactions (11). Nonetheless, there are subtle differences in the residues/interfaces that form the interaction and in the trajectory of pantetheine arms relative to the PCP domains when interacting with the alternate domains (Fig. S5). Although confirmed to be present in the protein prior to crystallization set up, no density was present to warrant inclusion of the tripeptide mimic in the final model. While the catalytically inactive enzyme was used for this structure, we note that our prior studies with SulTE C2818A mutant that showed no hydrolysis were tested only for 2 h. As crystals took ∼2 weeks to grow in preparation for harvesting for diffraction studies, there was ample time for slow peptide hydrolysis, whether spontaneous or specifically catalyzed by the mutant TE domain. We note also that we have previously examined a comparable cysteine to alanine mutant in the obafluorin system (39). Here, the assays were performed for longer time points, illustrating significant hydrolysis with the alanine mutant after 24 h. As such, the pantetheine is modeled as a free thiol.

**Figure 4:**
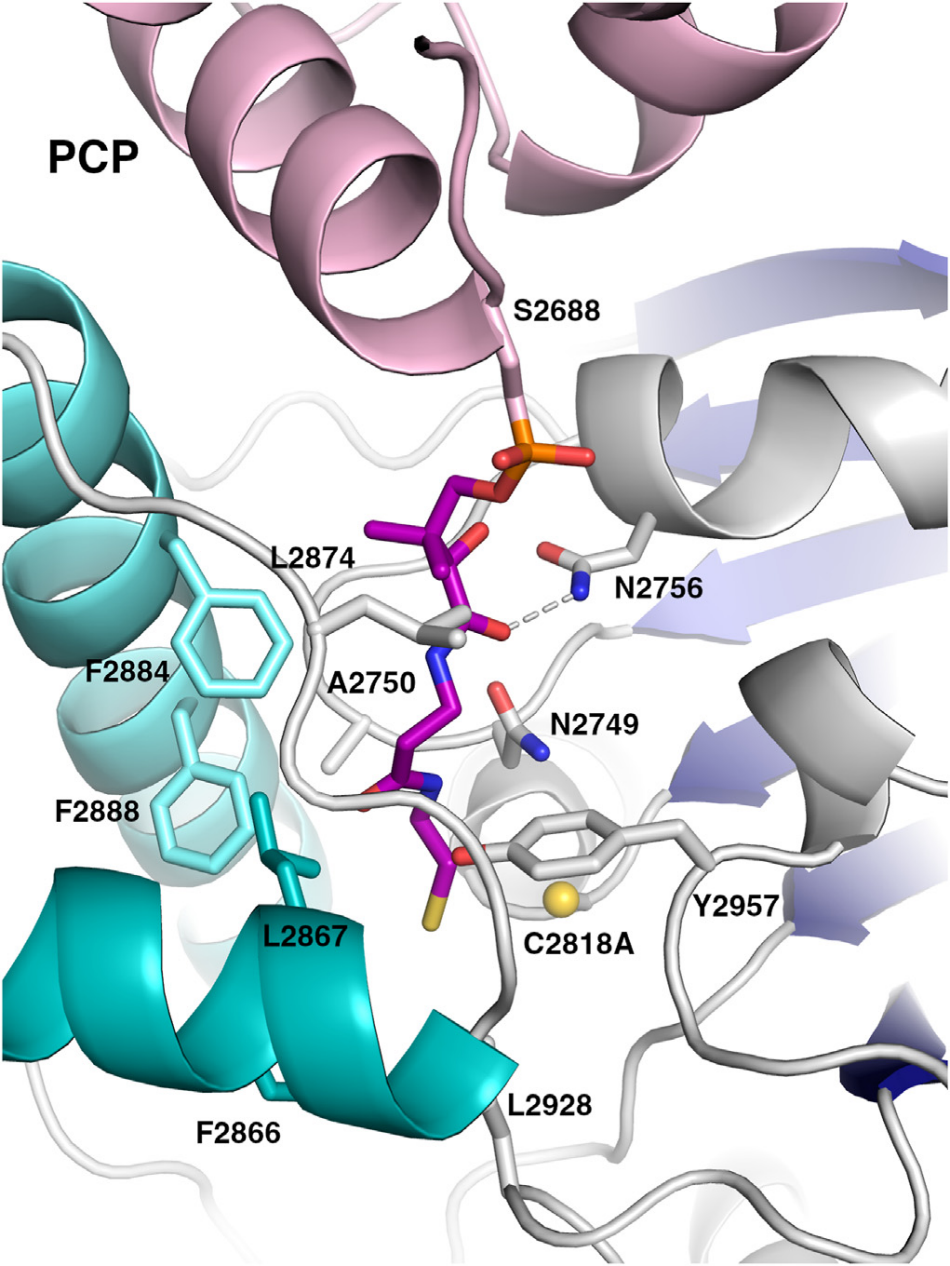
**Pantetheine tunnel of the SulM thioesterase active site.** The SulM thioesterase domain binds the PCP, allowing the pantetheine to pass through a mostly hydrophobic tunnel to enter the active site near the catalytic cysteine Cys2818, which has been mutated to an alanine for the complex structure.

Comparison of the structure of the isolated thioesterase domain with the didomain structure showed only small conformational changes in the lid helices. The rms displacement of the Cα positions in the TE domain is 0.5 Å (over 251 Cα atoms). All secondary structure elements superimpose well, with only the loop joining the αL1 and αL2 adopting a slightly different position in the didomain structure to increase the size of the pantetheine-binding tunnel (Fig. 4). The pantetheine reaches from the PCP domain into the active site, positioning the sulfhydryl 4 Å from Cβ of the alanine residue that replaced Ser2818. The pantoic acid dimethyl group of PPant interacts similarly in a hydrophobic pocket formed by Leu2874, Phe2884. The pantetheine hydroxyl group interacts with the side chain of Asn2756. The remainder of the pantetheine tunnel is rather hydrophobic, with Phe2866, Leu2867, Phe2884, Phe2888, Leu2928, and Tyr2957 all bordering the channel through which the pantetheine approaches the active site. Of note, the first four residues are located on the lid loop, illustrating its contributions to the functional interaction with the PCP.

### The interface between PCP and thioesterase domain

Acyl carrier protein domains generally contain four α-helices; three (α1, α2, and α4) are of similar length while α3 is shorter and lies perpendicular to the others (11, 44). The pantetheinylation site is located at the N-terminus of helix α2. To enable the pantetheine to approach the active site, the PCP domain binds into a cleft on the surface of the thioesterase domain. In the SulM didomain protein, two regions of the PCP are responsible for interactions with the thioesterase domain. A hydrophobic patch on helix α3 containing Val2704, Thr2705, and Tyr2708 interacts with a complementary hydrophobic region on the thioesterase domain composed of Met2733 on strand β2 and Pro2754, Val2755, and Val2758 that lie on the helix between strands β3 and β4. A second series of interactions occurs with PCP helix α2 that follows the pantetheine site. Phe2689 in the α2 of PCP interacts with a hydrophobic pocket formed by Leu2874, Pro2875, and Phe2884 in the TE domain, as well as the dimethyl group of the pantetheine cofactor. The PCP also forms polar interactions through Lys2690 and Arg2693 of α2 with Asp2879 and Gln2877 of TE domain.

To assess the conformational stability of the crystallized protein structures, we performed molecular dynamics simulations with both the thioesterase domain alone and the PCP–TE complex. The RMSD of SulTE (Fig. S6) and the SulM PCP-TE (Fig. S7) backbone atoms were calculated to analyze the conformational stability. An initial rise in the RMSD was observed for both proteins due to the relaxation in water environment. SulTE showed increased RMSD of 0.1 nm from 325 ns to 420 ns that returned to the average RMSD thereafter for the remainder of the 500 ns simulation. However, no fluctuations were observed in the SulM PCP-TE structure, which showed stable RMSD of less than 0.2 nm throughout simulation. Examination of the RMSF plots of both SulTE and the PCP-TE proteins (Figs. S6 and S7) indicated local flexibility at residues 2848 to 2884 representing the lid loop and lid helices 1 and several residues from lid helix 2. This is in agreement with NRPS TE domain crystal structures where lid helices and loop have been reported to show movement for substrate binding and play significant role in substrate recognition (14, 34). The SulM PCP-TE protein also showed local fluctuations for the loop connecting PCP and TE domains (residues 2721–2730). However, global fluctuations for PCP and TE domains or the residues at the interface were not observed indicating a stable interaction between two domains over the 500 ns simulation. Radius of gyration (Rg) analysis was performed to further understand the conformational dynamics and stability of PCP and TE domains. The SulM PCP-TE protein showed fluctuations lower than 0.1 nm throughout simulation run indicating the stable compactness of the protein and hence the PCP-TE conformation. Overall, MD simulation analyses support a stable conformation of individual PCP and TE domains and the observed interface of the PCP–TE complex.

### Comparison of the PCP–TE complex with EntF

**Figure 5:**
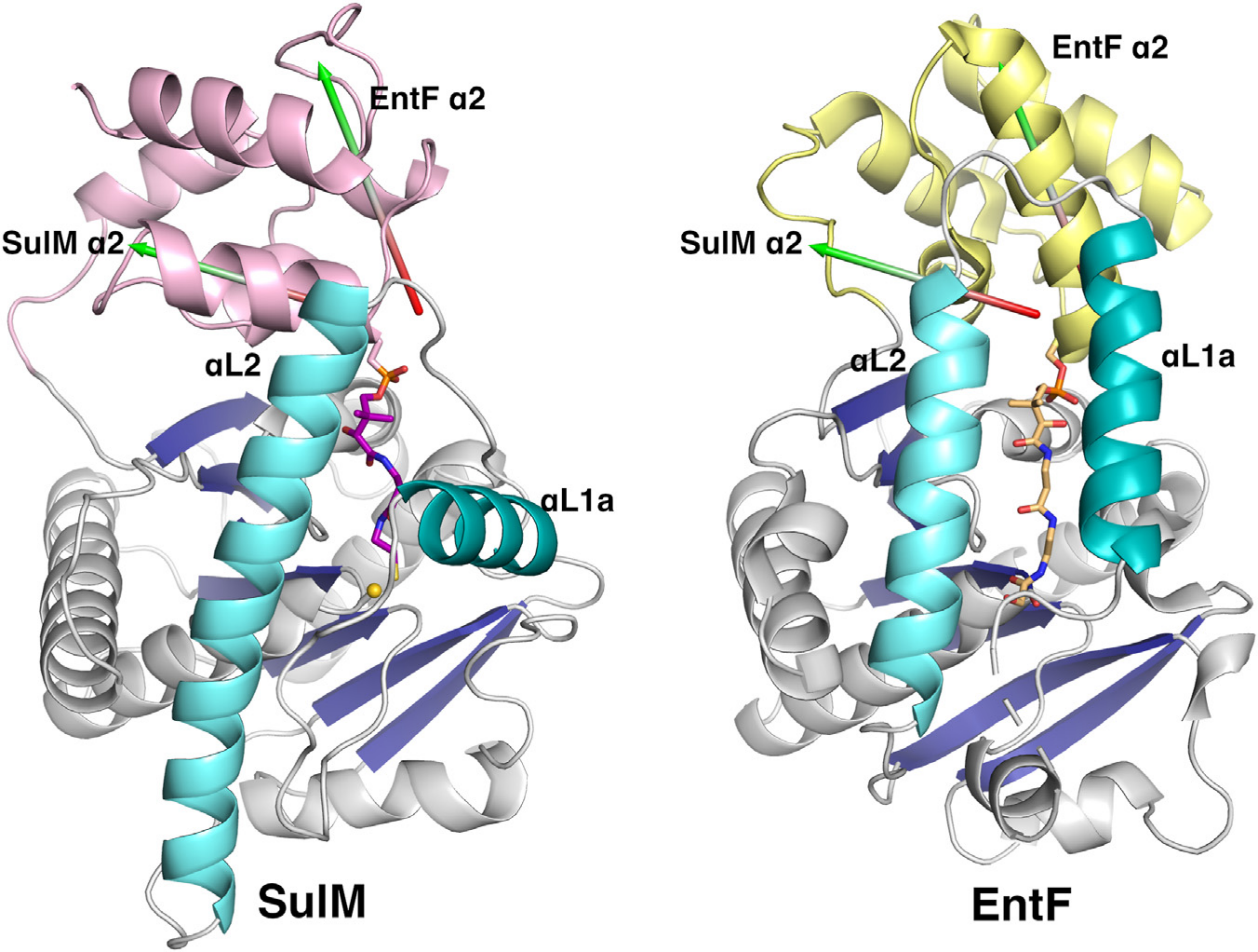
**Comparison of SulM_PCP-TE and EntF_PCP-TE interfaces.** The structures of the PCP-thioesterase didomain complexes were superimposed on the basis of the thioesterase domain, excluding the dynamic lid loops. The *holo*-PCP domains of each complex are represented in *pink* (SulM) or *yellow* (EntF, PDB 3TEJ). The PCP domains bind in the same region but adopt a different orientation. The α2 helix of each PCP is depicted with an arrow that is colored *red* to *green* in the N- to C- direction. The angle between the two helices is 50°, depicting the alternate positions adopted by the PCP domains.

To date, only the EntF thioesterase domain has been structurally characterized, both by NMR (15) and crystallography (16), bound to its carrier protein partner in a functional interaction. Both structures show a similar core thioesterase fold and interact with the PCP. The PCP of the NMR structure contains a mutation of the pantetheinylation site serine to an alanine, while the crystal structure contains an analog of the acyl pantetheine and thus sits deeper in the thioesterase domain pocket. The PCP–thioesterase complex from EntF was captured crystallographically using an α-chloroacetyl amino phosphopantetheine designed to stabilize the interdomain interaction (16). We superimposed the EntF and SulM thioesterase domains, allowing the comparison of the orientation of their respective PCP domain partners. The two thioesterase domains overlay with an rms displacement of 2.7 Å over all Cα positions excluding the flexible lid loops. The Cα positions of the serine residues from the two PCPs that harbor the pantetheine are 3.1 Å apart; however, the relative orientation of the two carrier domains differs. After superposition of the thioesterase core domains, the angle between the two α2 helices is 50° (Fig. 5). Examining the size of the interface, the EntF interface is more substantial, covering 915 Å^2^ in area. In contrast, the interface between the PCP and thioesterase domain of SulM is 516 Å^2^.

Despite these differences, the panthetheine moieties adopt a similar orientation to enter the active site. The sulfhydryl of the SulTE pantetheine and the corresponding amine of the pantetheine analog in the EntF structure (16) are 2.5 Å from each other. The lid loop helices appear to be a major cause of the alternate positioning of the PCP domains. The αL2 helices are similar in positioning although, as noted previously, the helix of SulTE extends several turns longer at the C-terminal end. The positions of the preceding helices, αL1 of SulTE and αL1b of EntF, adopt very different orientations, with the αL1b helix of EntF running parallel to the αL2 helix. The C-terminal end of the αL1b helix and the loop that connects it to the αL2 helix would clash with the PCP position adopted in the SulTE structure, thereby pushing it away from the active site to adopt the orientation seen in EntF.

### The active site of β-lactam/lactone-forming thioesterase domains enables proper orientation of the terminal nucleophilic residue

**Figure 6:**
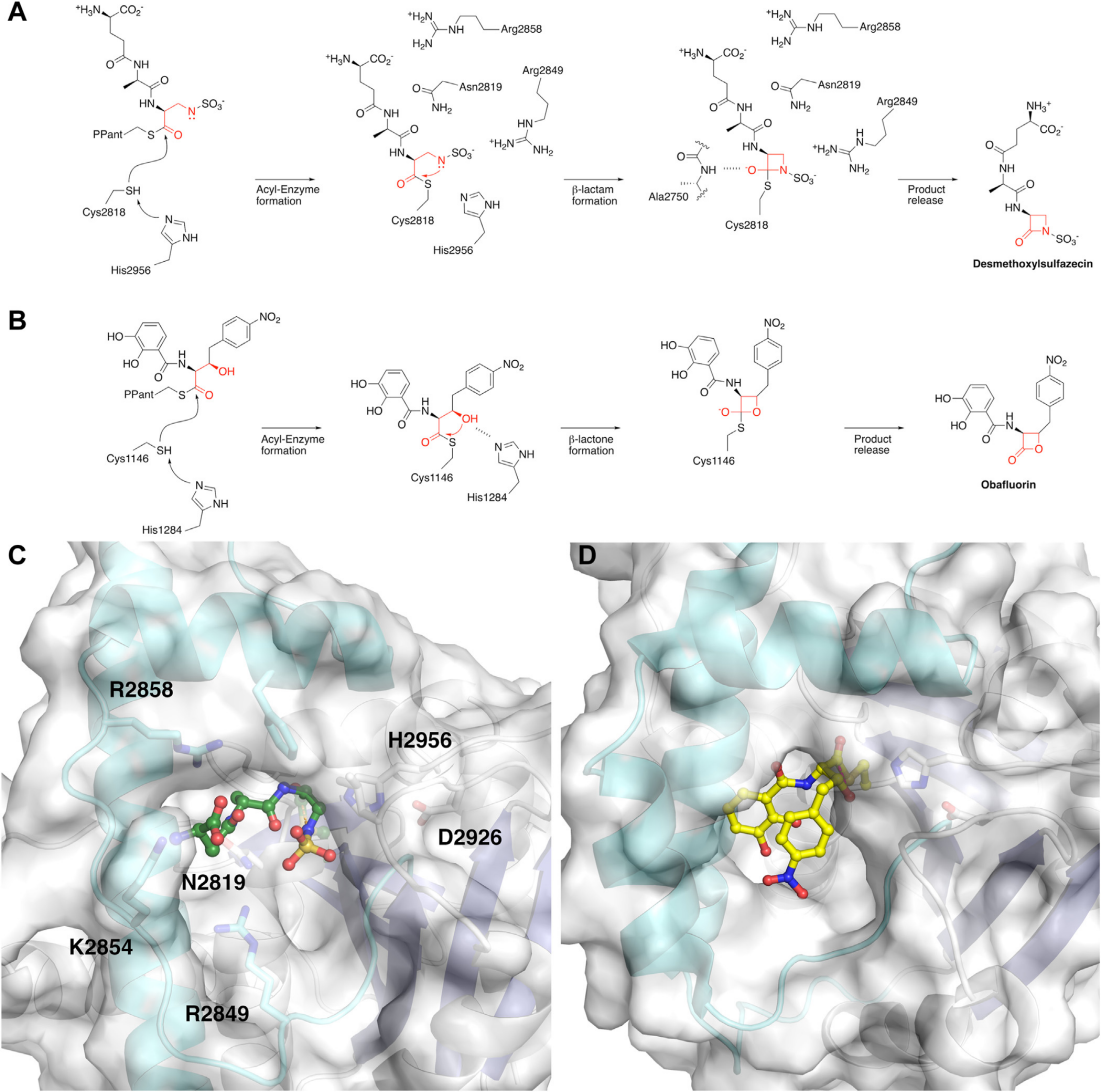
**The thioesterase domain creates a cavity for β-lactam/β-lactone formation.** Multistep reaction catalyzed by (*A*) the SulM thioesterase and (*B*) ObiF1 thioesterase domain. *C*, active site of the thioesterase domain with a modeled linear sulfazecin peptide that cradles the substrate for β-lactam formation. *D*, active site of the thioesterase domain of ObiF1 (PDB 6N9E) that positions the modeled obafluorin peptide for β-lactone formation. The view in *panel D* is rotated slightly around the Y-axis relative to *panel C*.

While macrocyclizations of NRPS products are relatively common, the SulM TE reaction poses the different challenge of forming the β-lactam ring. Biochemical evidence (23) shows that *N*-sulfonation by SulN precedes ring closure. Thus, to enforce 4-membered ring formation, the enzyme must position the *N-sulfo-*β-amine of the peptide in a constrained binding pocket to allow nucleophilic attack (Fig. 6*A*).

We modeled the sulfonated peptide into the active site with the goal of positioning the sulfonate nitrogen for attack and providing reasonable geometry for the remainder of the peptide given the tight fit of the active site (Fig. 6*C*). We modeled the peptide to interact with protein residues and to adopt torsion angles that are appropriate for a peptide and that position the sulfamate for β-lactam formation. In this configuration, the side chain of Arg2849 is directed towards the sulfamate SO_3_, directing the nitrogen for attack on the thioester carbonyl. The thioester carbonyl is directed toward the back of the pocket, enabling the main chain amine of Ala2750 to form part of the oxyanion hole that stabilizes the transient negative charge on the thioester oxygen during lactam formation. The sulfamate has a pKa of ∼8 (45) and we postulate that, further stabilized by Arg2849, a catalytic base is not required for activation. In this peptide orientation, the γ-glutamate carboxylate is near Lys2854 and Arg2858, while the amine is pointed at Asp2856. The main chain amide and carbonyl of the alanine residue from the sulfazecin tripeptide interact with Asn2819, the residue that follows the catalytic cysteine at Cys2818 (Fig. 6*C*).

Structure-based sequence alignment showed the Gln-Cys-Asn motif at the catalytic cysteine are present only in SulTE compared to other known thioesterase domain sequences from NRPS and PKS proteins (Fig. S8). In contrast to the SulM thioesterase domain, all other domains have a hydrophobic residue in place of Asn2819 and we propose that Asn2819 helps position the peptide for β-lactam ring formation. SulTE also shows a highly positively charged substrate-binding pocket required to accommodate negatively charged glutamate and sulfamate groups of the tripeptide substrate (Fig. S9).

We similarly modeled the active site of the ObiF1 thioesterase domain (39) containing the peptide poised for formation of the β-lactone (Fig. 6, *B* and *D*). The pocket is similarly highly constrained to position the β-hydroxyl of the *p*-NO_2_-β-hydroxyhomophenylalanine moiety for attack on the carbonyl of the thioester. As the β-hydroxy group will have a higher pKa, it is possible that His1284 may play its expected role as a catalytic base to activate the hydroxyl, in analogy to the role that the catalytic histidine plays in activating a water for hydrolysis in the bifunctional thioesterase domain of NocB in the nocardicin pathway (13). The ObiF1 thioesterase domain pocket is very hydrophobic in nature, simultaneously orienting the peptide while preventing access of water for hydrolysis of the thioester on the active site cysteine residue.

#### Phylogenetic analysis and structure-guided genome mining

A phylogenetic tree of thioesterase domains selected from the literature was generated using a Clustal Omega sequence alignment. The domain amino acid sequences were aligned with SulTE homologous sequences and a neighbor-joining phylogenic tree was constructed (Fig. S10). Surprisingly, the β-lactam and β-lactone–forming SulTE and ObiF1_TE grouped with type-II proof-reading domains in the phylogenetic tree, which also have the catalytic Asp residue shifted to position II after β7 strand.

In order to identify other clusters harboring SulTE-like β-lactam–forming TE domain, a PSI-BLAST search was performed using the SulTE sequence. Most of the hits with more than 70% similarity matched to SulM homologs from *Burkholdaria* bacteria with similar module organization previously suggested to synthesize sulfazecin (22). However, hits with lower than 70% sequence similarity identified clusters with different domain organizations in bacteria other than *Burkholdaria* and variable adenylation domain Stachelhaus and Challis code residues compared to the sulfazecin cluster (46, 47). To support the presence of β-lactam–forming TE domains within these clusters, we established four criteria based on SulTE structural features (Figs. S6 and S11). The initial criterion involved examining for the presence of a catalytic residues with aspartate at position II and the presence of the Gln-Cys-Asn motif housing the catalytic cysteine, allowing for interaction of the asparagine with the penultimate peptide residue. Additionally, TE domain sequences were distinguished by identifying positively charged residues at two out of three arginine positions, providing a positive active site environment to interact with the sulfamate. The presence of specificity-determining residues that match the 2,3-diaminopropionate activation in the adenylation domain of the last module was considered an additional criterion as the DAP residue is essential to form the β-lactam ring. Finally, the presence of a sulfotransferase gene in the cluster was also considered.

**Figure 7:**
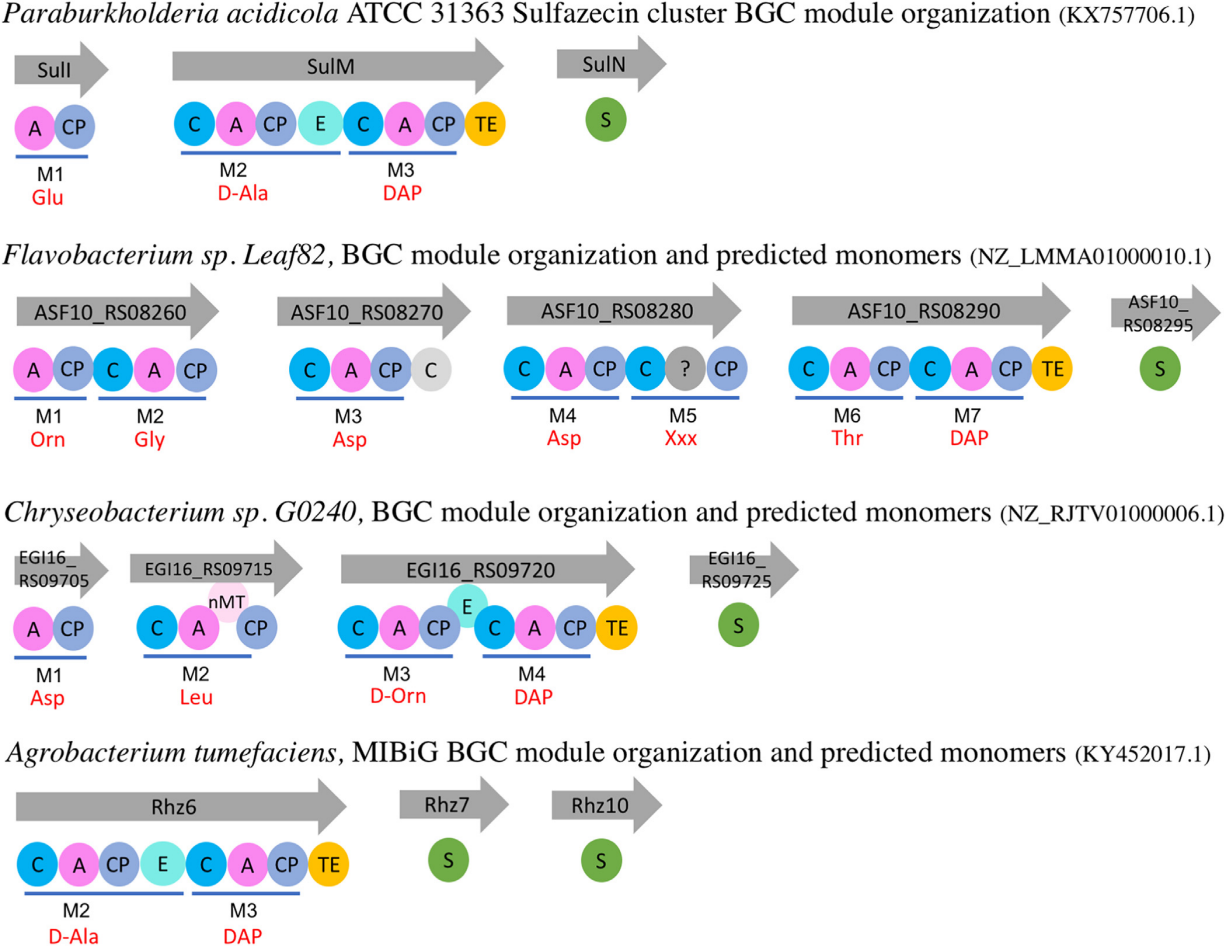
**Anti-SMASH analysis of likely new beta-lactam–forming clusters.** Anti-SMASH 7.0 was used to search for biosynthetic clusters (BGCs) using SulTE sequence as query in PSI-BLAST. The BGCs harboring SulTE-like domains were analyzed for module architecture and predicted substrates for each module. The predicted adenylation domain substrates differed from sulfazecin cluster except in last module which activates DAP required for β-lactam ring formation. Accession code for each cluster is provided in brackets and for each protein on the *arrow*. The predicted product and selectivity residues for the adenylation domain in the last module are shown in Table S3. Figure legends are as followed, A: adenylation domain, CP: carrier protein, C: condensation domain, C in *gray*: probable condensation domain, E: epimerization domain, TE: thioesterase domain, nMT: methyltransferase domain, ?: FkbH-like domain, S: sulfotransferase domain.

We highlight three clusters that varied from sulfazecin in the predicted amino acids activated by adenylation domains in upstream modules. On the basis of similar active site residues in the adenylation domain of the last module, these three clusters also most likely activate DAP for β-lactam ring formation. These clusters most likely biosynthesize peptides longer than sulfazecin, indicating the synthesis of novel β-lactam products. However, further investigation is required to confirm β-lactam formation by these clusters. A cluster from *Flavobacterium* sp. Leaf82 bacteria (Fig. 7) showed a large NRPS cluster predicted to be composed of seven modules. The upstream modules are predicted to produce an Orn-Gly-Asp-Asp-Xxx-Thr-DAP peptide. The fifth module showed the presence of an unknown domain in the place of an expected adenylation domain, which matched an FkbH-like domain upon BLAST search and most likely incorporates an unknown monomer. A second biosynthetic gene cluster from *Chryseobacterium* sp. G0240 contained four modules including an epimerization domain in the third module and is likely to place a D-ornithine preceding the β-lactam ring-forming DAP residue.

Finally, examination of the MiBIG repository (1) identified an unpublished β-lactam–containing peptide from *Agrobacterium tumefaciens* that showed similarity to the SulTE domain sequence and satisfied the structure-guided criteria of the thioesterase domain and the presence of a sulfotransferase gene in the cluster (https://mibig.secondarymetabolites.org/repository/BGC0001671/index.html). This example also strongly supports the use of the SulM thioesterase structural features to identify new β-lactam–harboring peptide products. Nevertheless, considering the differences in the sequence of the predicted peptide products, it is important to experimentally determine the identity and function of these peptides.

## Conclusions

We present here the structure of the monobactam-producing thioesterase domain from SulM in sulfazecin biosynthesis on its own and as a complex with the upstream *holo*-PCP domain. The structure illustrates that despite the unusual chemistry catalyzed by the SulM thioesterase domain, the overall fold of the protein is broadly shared with other structurally characterized NRPS thioesterase domains. While the core thioesterase domain fold is adopted by SulM, the dynamic lid helices reveal an unusual conformation seen in the β-lactone–forming thioesterase domain of the obafluorin biosynthetic protein, ObiF1 (39). We propose that the configuration of the lid helices results in a constrained active site that, upon formation of the acyl-enzyme intermediate, forces the *N*-sulfonated DAP moiety to adopt a geometry that is primed for β-lactam formation. This confined active site is also observed in the thioesterase domain of ObiF1.

The structure of the SulM thioesterase domain in complex with the upstream PCP is to our knowledge the second structure of the active complex between a PCP and the thioesterase domain. The complex is rotated ∼50° from the interface observed in the structure of EntF; however, both thioesterase domains direct the same face, the β2 strand, the helix that joins the β3 and β4 strands, and the lid helices towards the carrier protein and the trajectory of the pantetheine still allows a common approach to the active site.

Importantly, our structure-guided analysis identifies features that can be used to discover other NRPS-derived β-lactam antibiotics from previously uncharacterized biosynthetic gene clusters.

## Experimental procedures

### Cloning, expression, and protein production

The accession code for SulTE and PCP-TE sequence in SulM is AOZ21320.1. The protein sequences of the expressed constructs are described in Fig. S12.

The expression construct and purification strategy for PCP-TE, encoding Glu2660 through Ala2723, was previously described (23). The catalytic cysteine of the PCP-TE construct was mutated to an alanine. A final gel filtration run was used for structural analysis to remove aggregates and transfer the protein to storage buffer (20 mM MES, pH 6.0, 50 mM NaCl, and 0.5 mM triscarboxyethyl phosphine).

To produce an expression plasmid for the TE domain alone, multiple sites upstream of SulTE were screened for stable protein production. Oligonucleotides TE3-F and TE3-R were used to amplify the TE domain, which was initially cloned into pBluescript to give pBS/TE3.

TE3-F:5′-GGCATATGGCCTCGGAGGAGTCGAGCTCGATCGTG-3′

TE3-R 5′-GGAAGCTTTCTGCCGTCACACCTTTGCAGGACAC-3′

From pBS/TE3, the TE3 gene was excised with NdeI and HinDIII and ligated into pET28b. The final protein construct pET28 B/TE3 encoded residues Ala2723 through Ala2984 for the TE domain.

Proteins were expressed in *E.* coli Rosetta2 (DE3). An overnight culture was used to inoculate a large-scale growth, which was grown to A_600_ = 0.6 to 0.7 and cold-shocked on ice for 40 min before induction with 0.5 mM IPTG. The expression was carried out at 16 °C for 24 h. Cells were harvested by centrifugation. Ten grams of cells were suspended in 50 ml lysis buffer (50 mM NaH_2_PO_4_, 10 mM imidazole, 300 mM NaCl, 10% glycerol, pH = 8.0). Lysozyme was added to the final concentration of 2 mg/ml and incubated on ice for 30 min. The treated cells were subjected to sonication at 40% AMP for 13 min with 9.0 s/9.0 s pulse (Model GEX 400 by Ultrasonic Processor). The cell debris was removed by centrifugation at 41,400*g* for 25 min. The cell-free extract was mixed with 3 to 5 ml Ni-NTA resin and incubated at 4 °C for 60 min. The resin was washed 2 × with 10 to 15 ml wash buffer (50 mM NaH_2_PO_4_, 20 mM imidazole, 300 mM NaCl, 10% glycerol, pH = 8.0). The TE3 was then eluted with 3 ml imidazole elution buffer (50 mM NaH_2_PO_4_, 250 mM imidazole, 300 mM NaCl, 10% glycerol, pH = 8.0). A final purification step on a gel filtration column into storage buffer (50 mM MES, pH 6.0, 50 mM NaCl, and 0.5 mM triscarboxyethyl phosphine) was performed prior to crystallization.

### Attachment of sulfazecin tripeptide mimic to PCP-TE

The endogenous sulfazecin tripeptide was mimicked using L-glutamate in the place of L-DAP at the C-terminal position. The γ-D-Glu-D-Ala-L-Glu mimic was synthesized using conventional peptide chemistry to join D-Glu-D-Ala (23) to L-Glu. Peptidyl-mimic-S-CoA thioester was generated by coupling the tripeptide mimic to CoA (see supplementary methods). The CoA thioester was used to convert mutant *apo*-PCP-TE C2818A to its *holo*-form by combining with the peptide CoA thioester with 2 μM of the promiscuous phosphopantetheinyl transferase Sfp (23). The loading reaction was incubated at room temperature for 1 h (21), then buffer exchanged into buffer by three serial dilutions and concentrated using an Amicon Ultra 3K (Millipore). Loading of the peptide mimic was confirmed by high resolution MS (Fig. S13).

### Crystallization and structure solution

Initial hits for crystallization of both proteins were identified from a high-throughput sparse matrix screen (Hauptman-Woodward Institute) (48, 49). Further optimization of crystallization was performed in micro-batch under oil plates at room temperature. A drop of 1 μl of protein was added by 1 μl of crystallization cocktail in each well and the plate was overlayed with 5 ml of mineral oil. Data collection for both protein crystals were performed remotely at SSRL beamline 9-2.

Crystallization of the free thioesterase domain was performed at 10 to 12 mg/ml with a crystallization cocktail containing 100 mM Bis-Tris Propane pH 7.0, 100 mM ammonium bromide, and 40% PEG 20,000. Initial crystals were long thin rods nucleating from a single point. These crystals were used as seeds to grow diffraction quality crystals in the same conditions. Cryo-protection of crystals was done by serial transfer into crystallization cocktail supplemented with 10 and 20% NDSB mixture (60% ethylene glycol and 200 mg/ml NDSB-201, in water). Diffraction data were indexed, scaled, and integrated with HKL2000 in space group *P*2_1_. Structure determination was performed with Phaser in PHENIX using the ObiF1 (39) thioesterase domain (PDB 6N8E, residues 1059–1303) as molecular replacement model. The model was built and refined iteratively using COOT and PHENIX, employing translation-libration-screw parameters in the final stages of refinement.

The didomain PCP-thioesterase construct was crystallized at 10 mg/ml with a cocktail containing 1.8 M tribasic ammonium citrate, pH 7.0 to 7.5. Clusters of needle-like crystals were used as a seed stock to grow thin plate-like crystals. Further seed stock was made from these plate-like crystals and seeded into drops with different pHs from 7.0 to 7.5, made by mixing different ratios of stock solutions at pH 7 and 7.5. Crystals were transferred to fresh drops and diffraction quality crystals were obtained. Cryo-protection of crystals was done by transferring into mother liquor supplemented with 10% glycerol. From a single crystal that grew from a cocktail containing a 70:30 cocktail ratio of pH 7.0 and pH 7.5, diffraction data were collected, then indexed, scaled, and integrated with HKL2000 in space group *P*2_1_. The single domain SulM thioesterase structure was used as molecular replacement model for structure determination with Phaser in PHENIX. Model building and refinement was done iteratively using COOT and PHENIX. The PCP domain was built manually in COOT. The phosphopantetheine was modeled in the observed density and refined. Although loaded, the density for the peptide mimic was not of sufficient quality to be included in the model translation-libration-screw parameters, which were used during final stages of refinement.

### Molecular dynamics analysis of thioesterase and PCP-thioesterase structures

MD simulations for SulTE and SulM PCP-TE crystal structures described in the present study were carried out by Gromacs v2023.1 (50). Missing residues were fit into the structure by utilizing the partial density from experimental crystallographic data in COOT. Water and ligand molecules were removed from the crystal structures. Amber99SB-ILDN force field was utilized to generate the topology parameter files (51). SPC/E water model was used to solvate the proteins in a cubic simulation box of side 72 Å for SulTE and 85 Å for SulM PCP-TE (52). Both systems were neutralized by adding sodium ions. Energy minimization for each system was done using the steepest descent algorithm for 50,000 steps with a convergence tolerance of 150 kJ mol-1 nm-1. Systems were equilibrated under NVT-ensemble and NPT-ensemble states for 250 ps and 500 ps, respectively. Temperature coupling was done using the v-rescale thermostat at 300K (53). Pressure coupling was done using the Parrinello-Rahman barostat to maintain constant 1 bar pressure (54). A production run of 500 ns was performed with a 2 fs per step at 300K. The coordinates and trajectory were recorded at intervals of every 10 ps. Analyses of MD trajectories were performed by Gromacs utilities trjconv, rms, rmsf, and gyrate. Protein RMSD plots were generated using the MD Davis in Anaconda environment (55). Radius of gyration (Rg) and RMSF were plotted in Excel to assign residue numbers according to the deposited crystal structure.

#### Additional computational techniques

The superposition of SulM thioesterase domain with existing NRPS structures was performed with PYMOL using the super command with residues from the full domain lacking the lid loop spanning β6 and β7. The root mean square (rms) displacement is reported in the Supporting Information. The topology diagram was adapted from Horsman *et al.* (6) and was created with TOPDRAW (56). The angle between helices module of PYMOL is provided under open access license at: https://pymolwiki.org/index.php/AngleBetweenHelices.

The phylogenetic tree generation was calculated using structurally characterized thioesterase domain sequences, which were aligned in Clustal Omega. The phylogenetic tree was downloaded and visualized in iTOL (57). A bioinformatic search for potential β-lactam–forming clusters was performed by using the SulM protein sequence in PSI-BLAST search for homologous sequences. Hits lower than 70% similarity were analyzed for the presence of other NRPS modules in the cluster and the identification of the structure-guided sequence motifs and a sulfotransferase gene, as described in Results.

## Data availability

The atomic coordinates and structure factors used to solve the structures of SulM TE (8W2C) and the didomain SulM PCP-TE (8W2D) have been deposited with the wwPDB.

## Supporting information

This article contains supporting information.

## Conflict of interest

The authors declare that they have no conflicts of interest with the contents of this article.
