# Supplementary material for: The structure of the monobactam-producing thioesterase domain of SulM forms a unique complex with the upstream carrier protein domain[image]
Source: J Biol Chem. 2024 Jun 20;300(8):107489. doi: 10.1016/j.jbc.2024.107489 (PMC11298585; doi:10.1016/j.jbc.2024.107489)
Supplement: Supporting Information [file mmc1.pdf]

The structure of the monobactam-producing thioesterase domain of SulM forms a unique complex with the upstream carrier protein domain

Ketan D. Patel, Ryan A. Oliver, Michael S. Lichstrahl, Rongfeng Li, Craig A. Townsend, and Andrew M. Gulick

General Synthetic Methods ***S2***
Synthesis of γ-D-Glu–D-Ala–L-Glu-CoA Tripeptide mimic S3
Protected γ-D-Glu–D-Ala–L-Glu tripeptide benzyl ester 3 .... S3
γ-D-Glu–D-Ala–L-Glu-CoA Tripeptide mimic 4 .... S3
Supplemental Figures ***S4***
Supplemental Figure S1. Sequence and Structure Alignment of SulM thioesterase domain compared with prior NRPS thioesterase domain structures S4
Supplemental Figure S2. Comparison of Lid regions between different NRPS thioesterase domain structures S5
Supplemental Figure S3. Chemical structures of final products released from thioesterase domains of structurally characterized proteins S6
Supplemental Figure S4. The SulM PCP-thioesterase didomain structure illustrates the phosphopantetheine cofactor S7
Supplemental Figure S5. Phosphopantetheine arm orientations and interaction interfaces of PCP with NRPS domains S8
Supplemental Figure S6. Molecular Dynamics analysis of the SulTE protein S9
Supplemental Figure S7. Molecular Dynamics analysis of the SulPCP-TE protein S10
Supplemental Figure S8. Structure-based sequence alignment of thioesterase domains from NRPS and PKS proteins S11
Supplemental Figure S9. Electrostatic potential of SulTE and SulM_PCP-TE structure S12
Supplemental Figure S10. Phylogenetic tree of TE domain sequences from NRPS and PKS clusters S13
Supplemental Figure S11. Sequence alignment with SulTE domain homologous proteins S14
Supplemental Figure S12. Protein sequences of SulM thioesterase and PCP-thioesterase constructs S15
Supplementary Figure S13. Loading of sulfazecin tripeptide mimic to PCP3-TE C2818A S16
Supplementary Figure S14. ^{1}HNMR spectrum of Compound 3. .... S17
Supplementary Figure S15. Mass spectrometry of tripeptide-CoA thioester S18
Supplementary Figure S16. 1HNMR spectrum of Compound 4 .... S19
Supplemental Tables ***S20***
Table S1. Secondary structure of lid regions of structurally characterized NRPS thioesterase domains S20
Table S2: Crystallographic Diffraction and Refinement Data S21
Table S3. Potential uncharacterized β-lactam producing BGCs S22

General Synthetic Methods

All chemicals and reagents were purchased from Sigma Aldrich (St. Louis, MO), AK Scientific (Union City, CA), or Fischer Scientific (Hampton, NH), unless otherwise indicated, and used without further purification. All solvents were distilled before use, including THF (Na / benzophenone), DCM (CaH), and MeCN (4Å MS). Anhydrous DMF was purchased from Sigma Aldrich and used without further purification.

Preparative HPLC methods were carried out on an Agilent 1100 (Santa Clara, CA) series HPLC equipped with a multi-wavelength UV-Vis detector using one of the following HPLC prep methods:
HPLC Preparatory Method A: [binary gradient: water +0.1% TFA (solvent A), acetonitrile +0.1% TFA (solvent B), 4.5 mL/min]: 0-30 min gradient 0% to 100% B; 30-31 min gradient 90% to 10% B; 31-35 min isocratic 10% B. Phenomenex Kinetex® 5 µm C18 100Å LC column 250   10 mm.
HPLC Preparatory Method B: [binary gradient: water +0.1% TFA (solvent A), acetonitrile +0.1% TFA (solvent B), 4.5 mL/min]: 0-30 min gradient 0% to 60% B; 30-31 min gradient 90% to 10% B; 31-35 min isocratic 10% B. Phenomenex Kinetex® 5 µm C18 100Å LC column 250 10 mm.

UPLC-HRMS experiments to determine exact masses and purity levels of organic compounds were carried out on a Waters Acquity / Xevo-G2 UPLC-MS system at the Johns Hopkins Mass Spectrometry Facility. NMR spectra were recorded on either 400 MHz or 300 MHz Bruker (Billerica, MA) Advance NMR spectrometers. Chemical shifts are reported relative to the reference shift for the solvent used relative to TMS. Many 1H-NMR resonances are broad owing to amide and carbamate configurational equilibria. 13C NMR spectra were recorded on a 400 MHz Bruker Advance operating at 101 MHz. Chemical shifts are reported relative to the reference chemical shift of the NMR solvent. In 13C experiments for which D2O is the solvent, an internal standard of acetone was added, and the spectrum adjusted to this reference (215.94 ppm, 30.89 ppm).

Reagent abbreviations are as follows:

PyBOP: (Benzotriazol-1-yloxy)tripyrrolidinophosphonium hexafluorophosphate
DCM: dichlorormethane
DMF: *N,N*-dimethylformamide
DIPEA: *N,N*-diisopropylethylamine
MeCN: acetonitrile
TFA: trifluoroacetic acid

Thin layer chromatography (TLC) was carried out on silica gel coated glass plates with the elution conditions indicated, CV refers to column volumes of mobile phase used in silica gel chromatography, and tR indicates retention time.

Synthesis of γ-D-Glu–D-Ala–L-Glu-CoA Tripeptide mimic

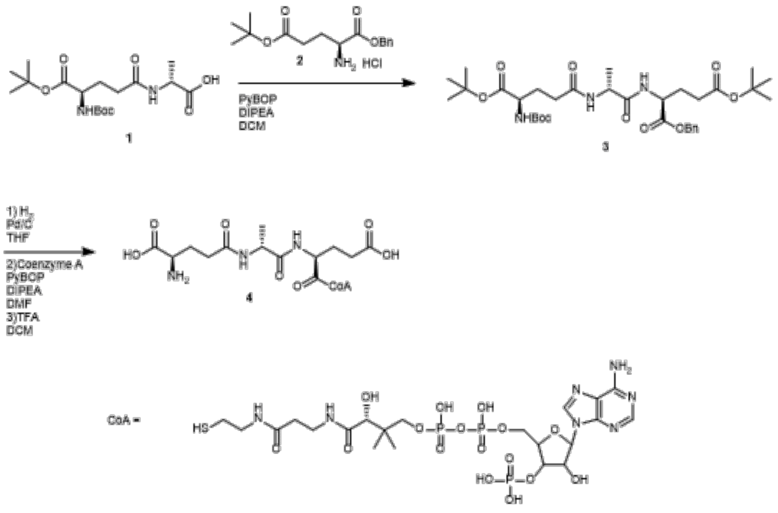

Protected γ-D-Glu–D-Ala–L-Glu tripeptide benzyl ester 3. Protected D-Glu-D-Ala dipeptide 1 (0.45 mol) was dissolved in 5 mL of DCM. To the solution was added doubly protected D-glutamate **2** (1.35 mmol) followed by PyBOP (0.54 mmol) and DIPEA (1.39 mmol) and the solution stirred overnight at room temperature. The organic phase was washed with aqueous $NaHCO_3$ and NH_4Cl, and the combined organics were dried over anhydrous $MgSO_4$. Protected γ-D-Glu-D-Ala–L-Glu tripeptide benzyl ester **3** (0.23 mmol 51 % yield) was isolated by silica gel column chromatography eluting with EtOAc/hexanes. **^{1}H-NMR** (400 MHz, $CDCl_3$): δ 7.38-7.28 (m, 5H), 7.03 (d, *J* = 7.8 Hz, 0.5H)6.49 (d, *J* = 6.5 Hz, 0.5H), 5.23 (d, *J* = 7.8 Hz, 0.5H), 5.18-5.08 (m, 2H), 4.61-4.53 (m, 0.5H), 4.54-4.45 (m, 0.5H), 4.16-4.08 (m, 0.5H), 3.49 (dd, *J* = 8.3, 5.2 Hz, 0.4H), 2.33 (t, *J* = 7.5 Hz, 1H), 2.29-2.20 (m, 2H), 2.20-1.74 (m, 4H), 1.56 (s, 11H), 1.44 (s, 5H), 1.42-1.39 (m, 13.5H), 1.36 (d, *J* = 7.0 Hz, 1.5H) **HRMS** (ESI) *m/z*: [M+H]+ $C_{33}H_{52}N_3O_{10}$ calculated 650.3647, found 650.3466

γ-D-Glu–D-Ala–L-Glu-CoA Tripeptide mimic 4. Protected γ-D-Glu-D-Ala-L-Glu tripeptide benzyl ester **3** (0.088 mmol) was dissolved in 10 mL anhydrous THF. Pd/C (5.7 mg, 10% wt/wt) was added and the solution stirred under balloon pressure hydrogen gas for 3 h at room temperature. The solution was then filtered through a 0.5 μM filter and the volatiles removed *in vacuo*. The resulting free acid peptide (0.063 mmol) was dissolved in 2.5 mL of anhydrous DMF. Coenzyme A sodium salt hydrate (0.069 mmol) and DIPEA (0.14 mmol) were added followed by PyBOP (0.075 mmol). The reaction was stirred under inert atmosphere of argon for 2 h at room temperature and then HPLC purified (HPLC method A) to yield protected γ-D-Glu–D-Ala–L-Glu-CoA tripeptide thioester. The lyophilized material was subsequently deprotected by stirring in 2 mL of a 3:1 TFA:DCM solution at room temperature for 1 h. The volatiles were then removed *in vacuo* and the reaction resuspended in 3 mL of ACN and purified by HPLC method B to yield γ-D-Glu–D-Ala–L-Glu-CoA tripeptide mimic **4** (0.0098 mmol, 11%). **^{1}H-NMR** (400 MHz, D_2O): δ 8.88 (s, 0.75H), 8.85 (s, 0.25H), 8.68 (s, 1H), 6.55 (d, *J* = 5.5 Hz, 0.25H), 6.44 (d, *J* = 5.5 Hz, 0.75H), 5.43-5.35 (m, 0.35H), 5.19-5.10 (m, 1.65H), 4.93-4.88 (m, 0.4H), 4.69-4.63 (m, 0.35H), 4.61-4.45 (m, 3H), 4.34-.428 (m, 1H), 4.25 (s, 1H), 4.15-4.08 (m, 1H), 3.92-3.84 (m, 1H), 3.72-3.66 (m, 2H), 3.61-3.55 (m, 2H), 3.29-3.22 (m, 2H), 2.82-2.74 (m, 2H), 2.74-2.64 (m, 4H), 2.52-2.39 (m, 3H), 2.24-2.12 (m, 1H), 1.68-1.61 (m, 3H), 1.18 (s, 3H), 1.07 (s, 3H). **HRMS** (ESI) *m/z*: [M+H]+ $C_{34}H_{56}N_{10}O_{23}P_3S$ calculated 1097.2448, found 1097.2446

Spectral characterization of synthesized compounds 3 and 4 are presented in Figures S14-16.

Supplemental Figures

Protein	PDB	SulM	SrfA-C	Vlm2	EntF	NocB	ObiF	FenB	SkyXY	AB3403
**SulM**	**8W2C**		21 %	20 %	22 %	21 %	25 %	19 %	19 %	17 %
**SrfA-C**	**2VSQ**	1.8 Å		22 %	22 %	23 %	19 %	34 %	21 %	22 %
**Vlm2**	**6ECE**	3.2 Å	1.4 Å		20 %	23 %	23 %	20 %	25 %	26 %
**EntF**	**3TEJ**	2.7 Å	2.0 Å	2.0 Å		22 %	24 %	26 %	28 %	25 %
**NocB**	**6OJD**	2.4 Å	1.4 Å	1.5 Å	1.6 Å		25 %	24 %	27 %	27 %
**ObiF**	**6N8E**	1.3 Å	1.5 Å	1.7 Å	2.6 Å	2.0 Å		21 %	23 %	21 %
**FenB**	**2CB9**	1.8 Å	1.1 Å	1.4 Å	1.9 Å	1.3 Å	1.6 Å		23 %	21 %
**SkyXY**	**7CRN**	2.9 Å	2.0 Å	1.7 Å	1.2 Å	1.2 Å	2.0 Å	1.6 Å		25 %
**AB3403**	**4ZXI**	2.4 Å	1.5 Å	1.4 Å	1.5 Å	1.2 Å	1.9 Å	1.5 Å	1.4 Å	

Supplemental Figure S1. Sequence and Structure Alignment of SulM thioesterase domain compared with prior NRPS thioesterase domain structures. Values above the diagonal represent pairwise sequence identity of the thioesterase domains, calculated with CLUSTAL OMEGA. Values below the diagonal represent rms displacement by superimposing the core thioesterase domain with PYMOL super algorithm, lacking the dynamic lid loops

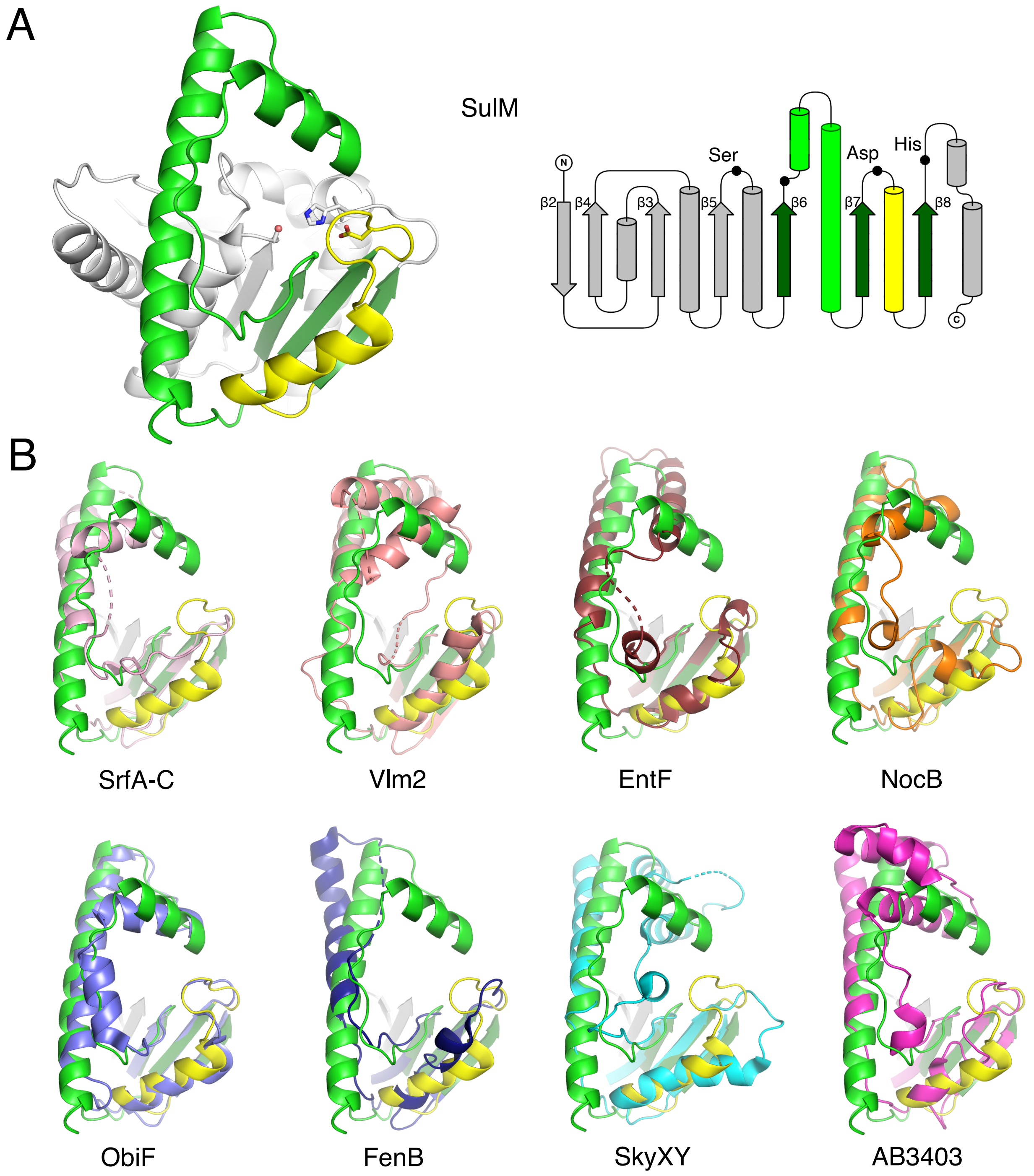

Supplemental Figure S2. Comparison of Lid regions between different NRPS thioesterase domain structures. A. Ribbon diagram of SulM thioesterase. The side chains of the residues that form the catalytic triad are shown. The pink sphere reflects the use of the C2818A mutant enzyme. The green sphere in the ribbon and the unlabeled black circle in the topology diagram highlight the more common position of the Asp in NRPS thioesterase domains. Topology diagram created with TopDraw and adapted from Horsman *et al*. (6). B. NRPS thioesterase domains (unique colors) were superimposed on SulM via the core of the protein. The overlay depicts the strands β6-β8 and the lid loop (light green) and the loop following β7 (yellow).

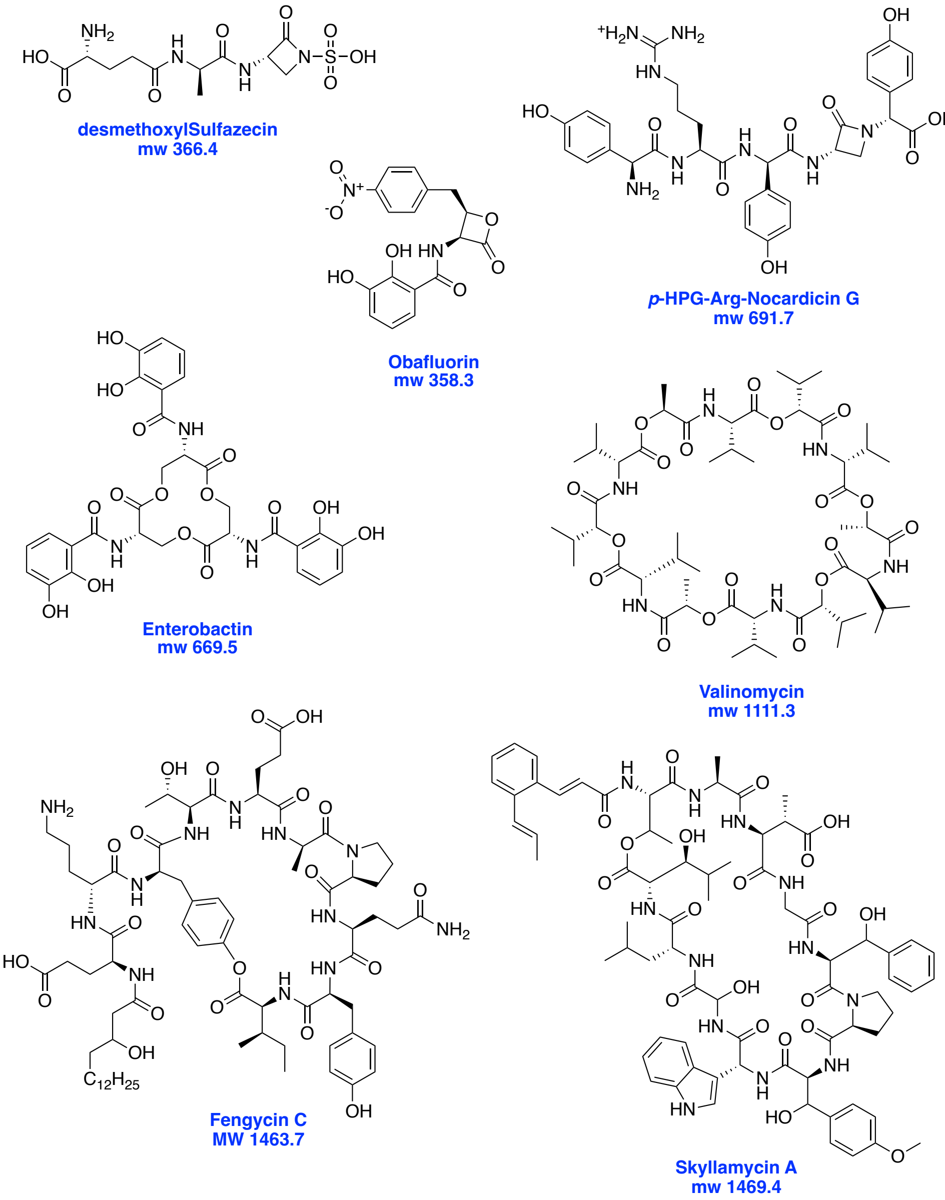

Supplemental Figure S3. Chemical structures of final products released from thioesterase domains of structurally characterized proteins. Release products of NRPS systems for which TE domain structure has been solved. In some cases, the product is further modified following release to yield the ultimate natural product. The structures and molecular weight do not include any chemical modifications that occur following release of the NRPS peptide.

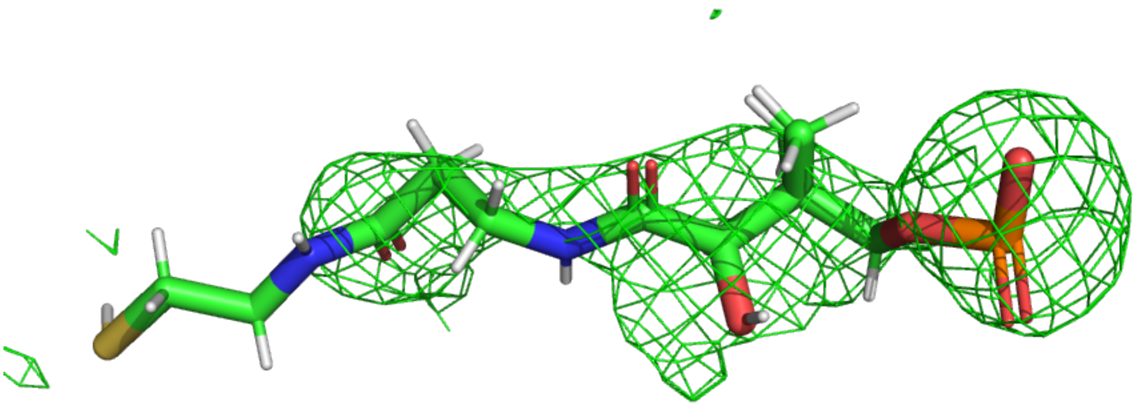

Supplemental Figure S4. The SulM PCP-thioesterase didomain structure illustrates the phosphopantetheine cofactor. Omit map electron density of the phophopantetheine cofactor was produced by removing the phosphopantetheine group and submitting the final structure to a single round of simulated annealing refinement. The map is calculated with coefficients of the form Fo-Fc and contoured at 2.5σ.

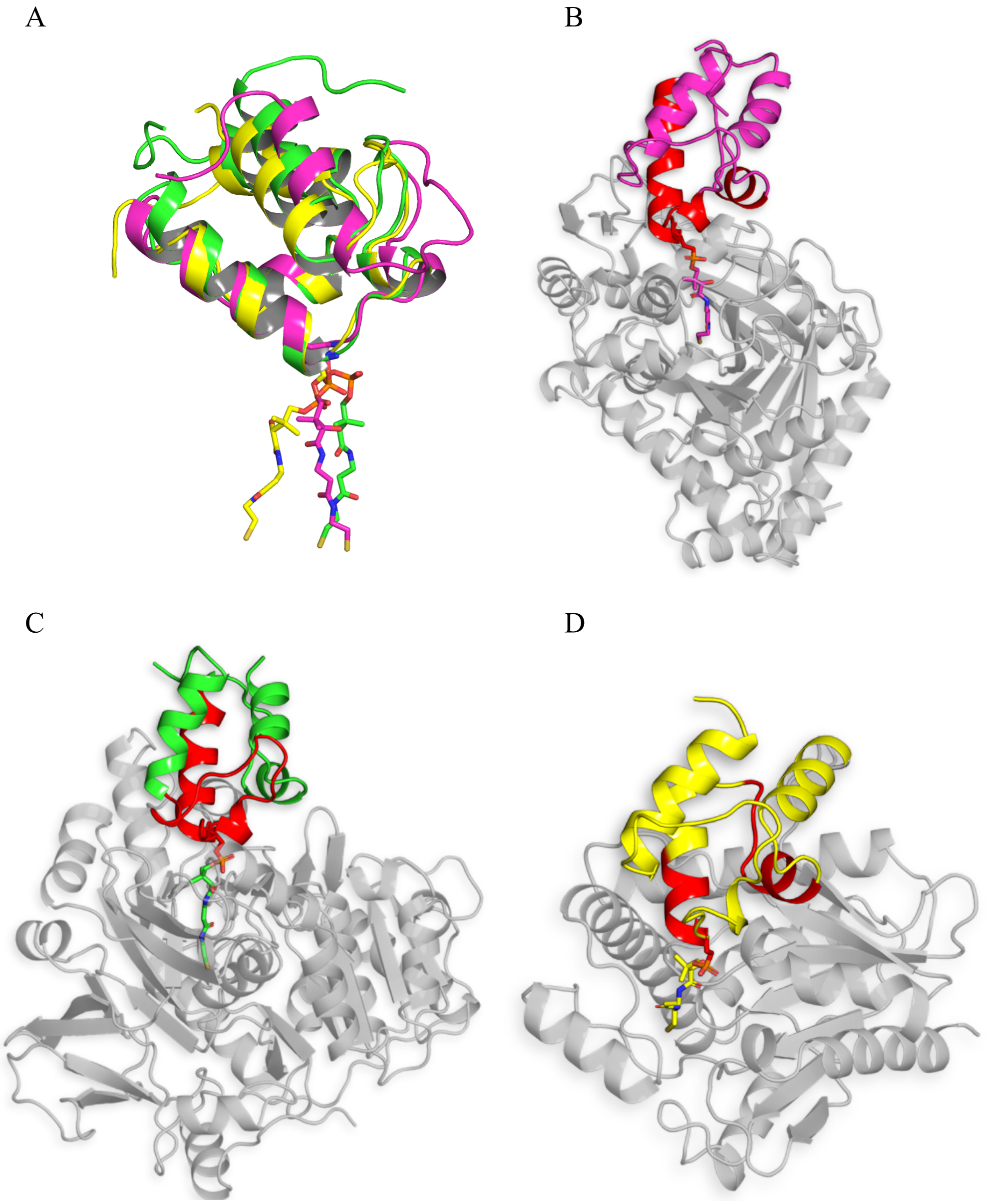

Supplemental Figure S5. Phosphopantetheine arm orientations and interaction interfaces of PCP with NRPS domains. A, orientations of phosphopantetheine arm to approach the neighboring active sites in adenylation domain (pink, PDB **3RG2**), condensation domain (green, PDB **4ZXI**), and SulM thioesterase (yellow, PDB **8W2C**). Interfaces of carrier domain (red) while interacting with B. adenylation domain (pink, PDB **3RG2**), C. condensation domain (green, PDB **4ZXI**), and D. SulM thioesterase (yellow, PDB **8W2C**), highlighting the orientation of the cofactor adopts to approach the neighboring active site. The PCP interactions with three catalytic domains employ different regions of the carrier domain.

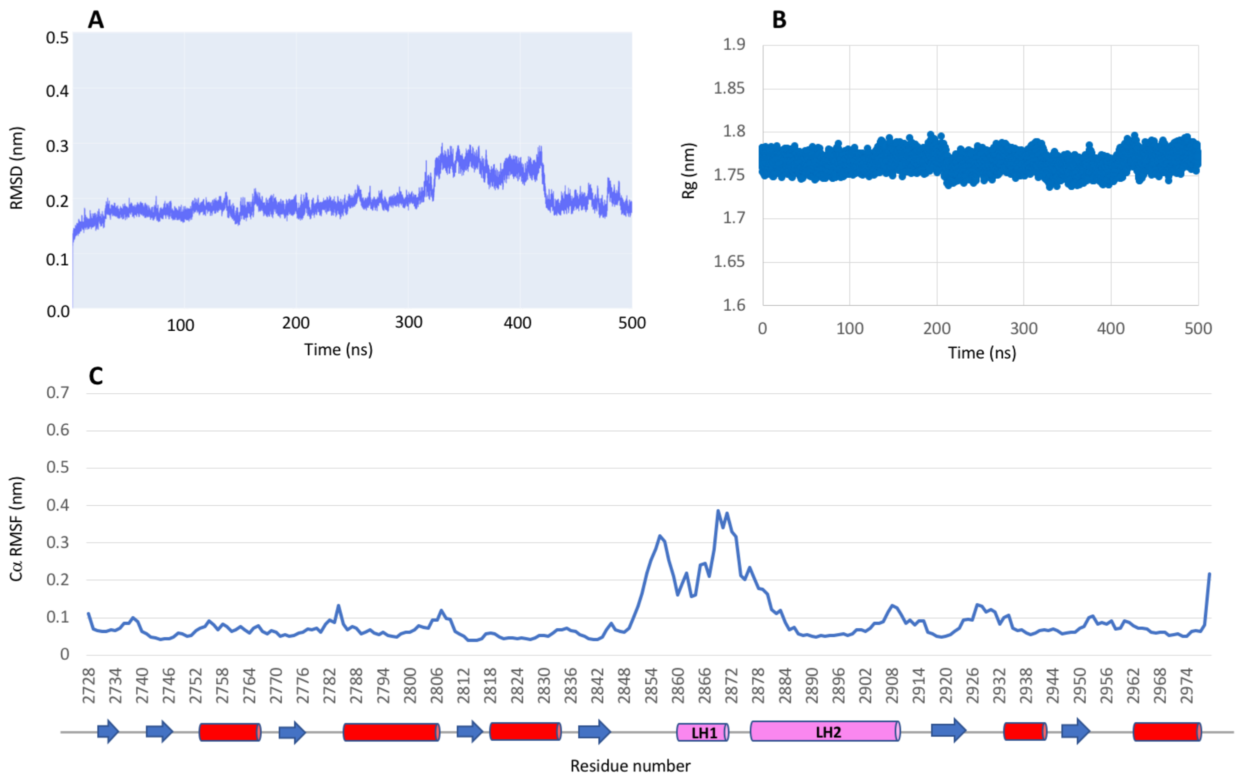

Supplemental Figure S6. Molecular Dynamics analysis of the SulTE protein. A) RMSD (root mean square displacement) plot of protein atoms, B) Radius of gyration (Rg) plot and C) Cα RMSF (root mean square fluctuation) plot for each residue calculated from the 500 ns simulation. Cylinder, arrow, and line represent the secondary structure elements of α-helix, strand, and loop respectively. Pink colored cylinders represent lid helix 1 and 2 (LH1 & LH2) of the thioesterase (TE) domain are labeled.

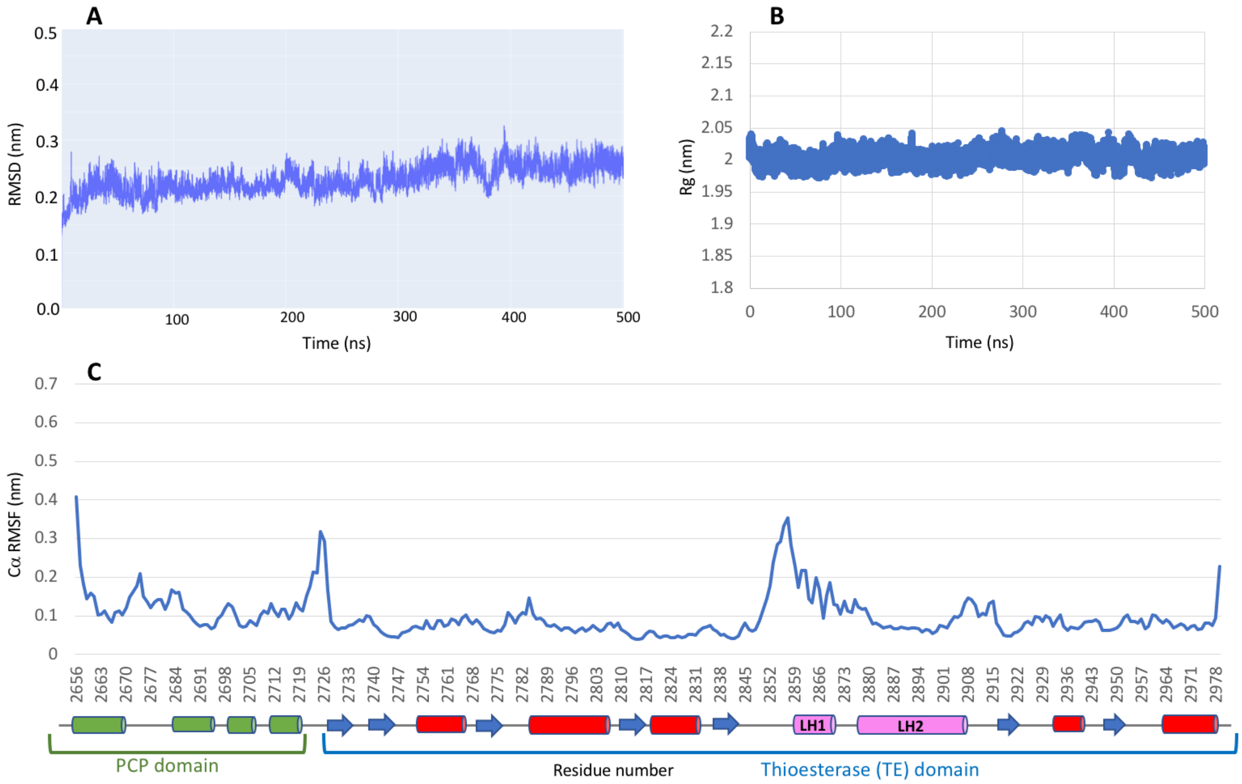

Supplemental Figure S7. Molecular Dynamics analysis of the SulPCP-TE protein. A) RMSD (root mean square displacement) plot of protein atoms, B) Radius of gyration (Rg) plot and C) Cα RMSF (root mean square fluctuation) plot for each residue calculated from the 500 ns simulation. Cylinder, arrow, and line represent the secondary structure elements of α-helix, strand, and loop respectively. Green colored cylinders represent the PCP domain α-helices, while pink colored cylinders represent lid helix 1 and 2 (LH1 & LH2) of the thioesterase (TE) domain.

```
FengTE_2CB9__             42  ALQLN---HKAAVYGFHFI---------------EEDSRIEQYVSRITEIQ-PEGPYVLLGYSAGGNLAF   92
2cb9_chainA_p007          36  ALQLN---HKAAVYGFHFI---------------EEDSRIEQYVSRITEIQ-PEGPYVLLGYSAGGNLAF   86
7VJT_                     46  LGRLP---ADTPMYGFERVE-------------GSIEERAQQYVPKLIEMQ-GDGPYVLVGWSLGGVLAY   98
7vjt_chainA_p006          39  LGRLP---ADTPMYGFERVE-------------GSIEERAQQYVPKLIEMQ-GDGPYVLVGWSLGGVLAY   91
6BA8                      38  QAEQL---ADCALSLVTWPGRDRLRHL---EPLRSITQLAALLANELEASVSPDTPLLLAGHSMGAQVAF  101
TycC_TE                   20  AAEIQ----GVSLYSFDFI---------------QDDNRMEQYIAAITAID-PSGPYTLMGYSSGGNLAF   69
SrfAC_TE                  20  SSRLP----SYKLCAFDFI---------------EEEDRLDRYADLIQKLQ-PEGPLTLFGYSAGCSLAF   69
RifR_                     40  AKALA---PAVEVLAVQYPGRQDRRHE---PPVDSIGGLTNRLLEVLRPFG--DRPLALFGHAMGAIIGY  101
3flb_chainA_p003          36  AKALA---PAVEVLAVQYPGRQDRRHE---PPVDSIGGLTNRLLEVLRPFG--DRPLALFGHAMGAIIGY   97
6VAP_                     44  AAALA---PRCDVLAVQYPGRQDRRAE---KPLEDIDELANQLFPVLRARV--HQPVALFGHSMGATLAF  105
6FVJ_                     48  SREFS---ADVKRIAVQYPGQHDRSGL---PPLESIPTLADEIFAMMKPSARIDDPVAFFGHSMGGMLAF  111
7E3Z_                     43  AEDLP---EDVELALICYPGREARFGA---PFARVWTELRDDVVRSVRGLT--GRPYILFGHSMGSWMAF  104
3QIT_                     46  ALPLA--AQGYRVVAPDLFGHGRSSHLE-MVTSYSSLTFLAQIDRVIQELP--DQPLLLVGHSMGAMLAT  110
TrdC                      38  ASGLG---DLLPCSIWETIPQEAG-------TGAGDADPVERWLEEVAADG--RPVRAVFGFCVGGVYAG   95
LipX2                     47  GRLLA---RGVPLWETRQPEPEQA-------RTFGGEDFASYWVRGVRDTG--RPVRAVLGYCVGGLYAA  104
KSE_70420                 39  LPHLN---PDFSLWQTVAPVRAPE-------TGAPPEEYLAAWLSEIEASG--RVVRAVMGYCAGSVFAG   96
KirH1                     39  TPSLG---LDCALWQTVAPARAPG-------PGIDPDEYLAPWLAEVAASG--RRVRAVLGYCAGSVFAG   96
SlgL                      53  VPNLP---ADRTVWETTQPALGQE-------TEMGGAAYVARWMRAVTESG--RYVRAVMGYCVGSVFAA  110
3QMV_                     61  QERLG---DEVAVVPVQLPGRGLRLRE---RPYDTXEPLAEAVADALEEHR-LTHDYALFGHSXGALLAY  123
7R0X_                     70  AENLG---DDYAIWGIHSRGFLTRH-----PPLKSIESXANYYIEILQAGN-ATGPYELAGYSXGGIIAY  130
NocTE                     39  APLLP----DVRLHVLSDPRFGQSD-----NRFATLAEMATRYVEWVRTTE-PEGPYRLGGWSFGGVVAL   98
6ojd_chainA_p005          32  APLLP----DVRLHVLSDPRFGQSD-----NRFATLAEMATRYVEWVRTTE-PEGPYRLGGWSFGGVVAL   91
3LCR__                    81  AEELD---AGRRVSALVPPGFHGGQ-----ALPATLTVLVRSLADVVQAEV-ADGEFALAGHSSGGVVAY  141
FtdB_TE_                  89  AGALR---GIAPVRAVPQPGYEEGE-----PLPSSMAAVAAVQADAVIRTQ-GDKPFVVAGHSAGALMAY  149
Erya3_TE                  21  AGALR---GIAPVRAVPQPGYEEGE-----PLPSSMAAVAAVQADAVIRTQ-GDKPFVVAGHSAGALMAY   81
MlaG_TE__                 22  GSEFR---DVRPVSALALPGFQRGE-----PLPESVEVLSQVLGEAVLAAA-DGDPYVLLGYSSGGIIGH   82
HitP3_TE_                 22  AAHFR---GVRPVSALPLVGFARGD-----LLPATAAAAAQVVAENVLRAA-GGEPFVLVGYSSGGVLAY   82
VinP4_TE                  22  AAHFR---GVREVSALPMPGFATGD-----LLPATAEAVVEVFGRSLLRAA-GDEPFVLLGYSAGGVFAH   82
PikTE                     91  STSFQ---EERDFLAVPLPGYGTGTGTGTALLPADLDTALDAQARAILRAA-GDAPVVLLGHSGGALLAH  156
TaaE_TE2                  20  TDAFG---PTWPIHGLQPRGLDAKA-----VPHSRVESAAQAYLVALEQQC-PSGPVHLLGHSFGGWVAF   80
MassC_TE2                 20  SEALG---RDWPLYGLQPRGLDGEA-----VPHSQVEAAAQCYLAALEQEC-PEGPVHLVGHSFGGWVAL   80
LybB_TE2                  20  TQACG---AHTPIIGLQARGHDGRS-----VPHASVEAAVEALLPEVRALG-QGGPYHLLGHSFGGWIAF   80
6ECB_                     39  ARHLK---GFGEIHGVEAPGLGAGET----PVYPSFEEMVQFCSDSAAGVA--GDGVYIGGHSLGGHIAF   99
BdObiF_TE                 34  ARALP---DGFACSALQLPGHDPAAPD---EAFVDLDTTIDRAVDRLLAEA--AAPIVVYGHCAGNALAV   95
BdObiF_TE.pdb_chainA_s001 34  ARALP---DGFACSALQLPGHDPAAPD---EAFVDLDTTIDRAVDRLLAEA--AAPIVVYGHCAGNALAV   95
LnmJ_TE_                   5  FAALS---ERYRVIVVHHPGVGDTTA----CEELGYEGIADLCLRALRRLG-VQGPVHVAGASFGGITAQ   66
3ILS_                     41  PR-LK---SDTAVVGLNCPYARDPE-----NMNCTHGAMIESFCNEIRRRQ-PRGPYHLGGWSSGGAFAY  100
3ils_chainA_p004          41  PR-LK---SDTAVVGLNCPYARDPE-----NMNCTHGAMIESFCNEIRRRQ-PRGPYHLGGWSSGGAFAY  100
SulM_TE                   45  SRAMPEQASDVAMFGVKLPRTEVDSDG---AMLEEVRRLSNAVCDDLLAAT--DLPAIVFAQCNGSALAL  109
SSHG_TE                   20  AAQLP----EYRVVAFNYLP---------------GDDKVARYADLVEAAR-PEGACRLLGYSLGGNLAF   69
FtdB                     138  ATSLP----SHRLIAFNYLP---------------GDDKVSRYADLVAATV-PEGPVPLLGYSLGGNLAF  187
SGR814_TE                 20  AAQLP----EYAVIGFNYLP---------------GDDKVARYADLIEAAR-PEGACLLLGYSLGGNLAF   69
Hsaf                      20  AVHLP----EYEFVSFNYLL---------------GDDKVARYADLIEGIH-AEGHCTLFGYSLGGNLAF   69
IkaA                      20  AARLP----EFEFLAFNYLM---------------GEDKVSGYADLVAGHR-PEGEIDLLGYSLGGNLAF   69
TaaE_TE1                  20  GQHLD---ADIPVYGLEGVAWGE-------PQLQTMECLAARHIDIMRGVQ-PHGPYRLAGWSFGGVLAY   78
MassC_TE1                 20  GQHLP---GDYPIYGLPGVALGE-------AHLDSMEGLAARMVGLIRQIQ-PHGPYRLAGWSFGGVLAY   78
LybB_TE1                  20  SRHFD---DDLPIYGIQADRADA-------QREVSAQSLARRYLEVIRSVQ-PQGPYRLAGWSGGGHLAY   78
HbnA                      20  AVHSR---SAEPLAAVSYPGEED-------GRPGTLRELAALYVEQIRREQ-PHGPYRLGGYSFGGNVLF   78
2K2Q_                     33  HAFLQ---GECEMLAAEPPGHGTNQT----SAIEDLEELTDLYKQELNLRP--DRPFVLFGHSMGGMITF   93
7CRN_                     43  MQHIG---ADRPLYGLQARGLADPSA----TLPSSIEEMAADYVTQIRGVQ-PSGPYHLLGWSLGSLVIH  104
Pys-Pflu_TE               20  LPFLP---EDQPMYALQSPILRDPT-----RVIGSLDELAAEYLQRIVDLH-PEGPYQLAGWSVGGNLAL   80
Pys-Pent_TE               20  LAYLP---QDQPLYALQSPILLDPE-----RVVGSLEELAREYLQRILALQ-PQGPYQLAGWSVGGNLAL   80
EntF_TE                   34  SRYLD---PQWSIIGIQSPRPNGPM-----QTAANLDEVCEAHLATLLEQQ-PHGPYYLLGYSLGGTLAQ   94
AB_TE                     32  SRHLN---PNRTLRAIQSPGLIEAD-----AAEVAIEEMATLYIAEMQKMQ-PQGPYFLGGWCFGGAIAY   92
AB_TE.pdb_chainA_s002     32  SRHLN---PNRTLRAIQSPGLIEAD-----AAEVAIEEMATLYIAEMQKMQ-PQGPYFLGGWCFGGAIAY   92
SwrW_TE                   20  ANYLE---KDFNVLGINNYNLFQQ------PHITSLRELAAYYLGHMRLS--PARPVRLLGWSLGGLIAL   78
5UGZ_                     30  RSVLS---DNITLRPLEPAGRGTRIRQ---PLCLTMVDAVADLYQQFVKHY-TGGDYAIFGHSLGGIMAF   92
Consensus_aa:                 ...h........h.shp.................s...hh..hh..l......p.sh.lhG@thGt.hAh
Consensus_ss:                 hhh      eeeee                   hhhhhhhhhhhhhhh      eeeeee hhhhhh
```

Supplemental Figure S8. Structure-based sequence alignment of thioesterase domains from NRPS and PKS proteins. Green box shows alignment of "QCN" residues of SulTE domain with other TE domain sequences. Presence of "QCN" residues was only found in SulTE sequence. PROMALS3D server was used for alignment. (http://prodata.swmed.edu/promals3d/results/phpONqyVB.result.html)

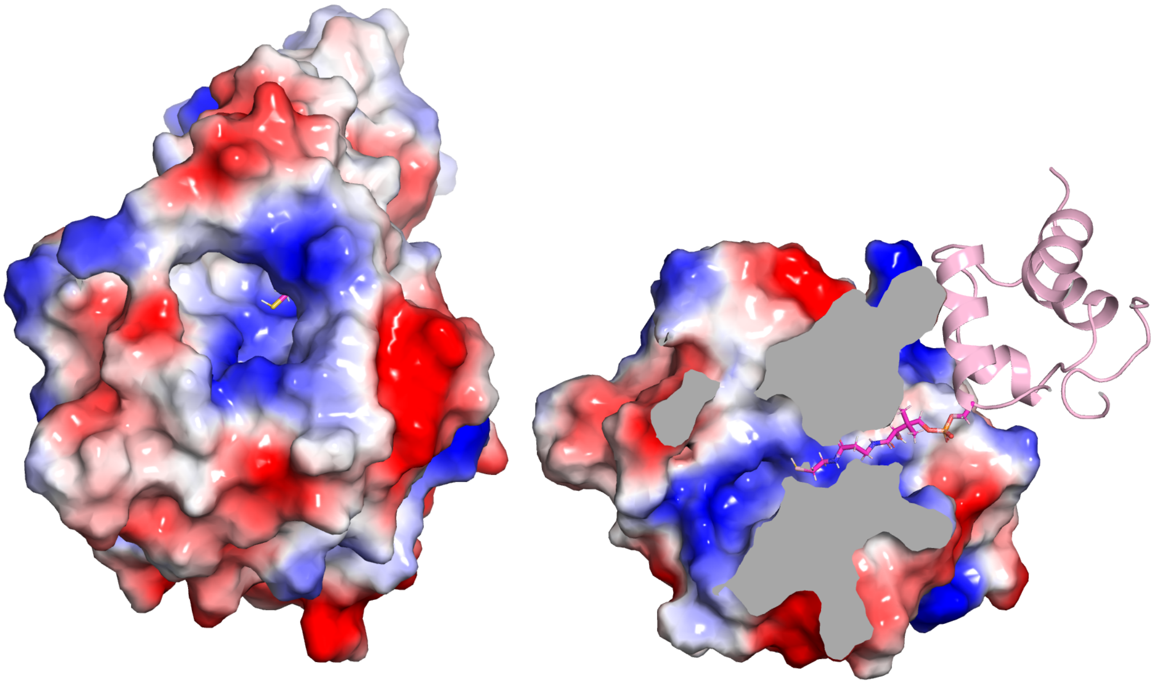

Supplemental Figure S9. Electrostatic potential of SulTE and SulM_PCP-TE structure. Positively charged substrate binding pocket at the center in SulM_PCP-TE (left) which likely favors binding with negatively charged tripeptide. The phosphopantetheine arm approaches positively charged substrate binding pocket through a tunnel in the SulTE domain (right)

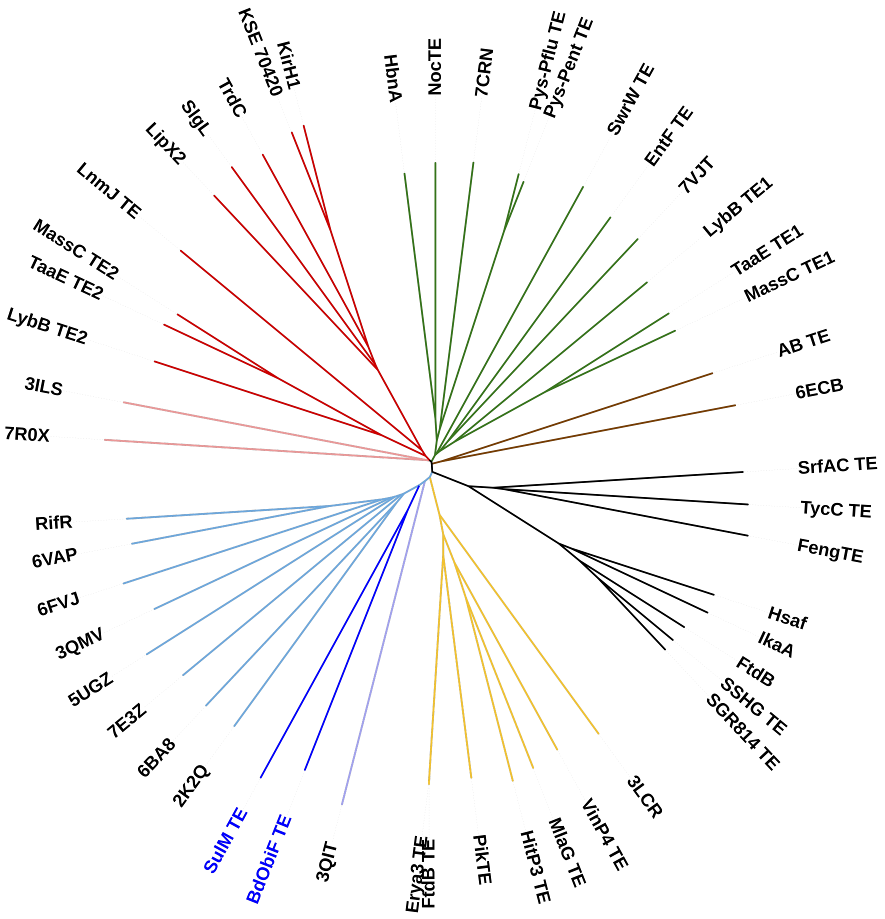

Supplemental Figure S10. Phylogenetic tree of TE domain sequences from NRPS and PKS clusters. The Interactive Tree of Life software was used to create a phylogenetic tree of thioesterase domains.

```
AQX14441.1_Agrobacterium         ---------------------LHLLSAEEDVGRILVPVAGDLSSAKVRIVAVSNSGGDPI 39
SulM_TE                          ---------------------MEHLEHASEESSSIVLMAGDPATAKAVVVCVANAAGGPV 39
WP_081284758.1_Chromobacterium   -------------------------------QKTLTPIAGDPVQAEAMLLCIANSGGGPV 29
WP_088511513.1_Pseudomonas       ----------------------------STERRVLTRICGDAADADAVFVCIANSGGGPV 32
WP_056247584.1_Flavobacterium    VSLSDSYTVPDLYRNPTIRELAKHIKENKKSNDLLTKMLDVKEKAEVTIIGIPNSAGDPL 60
WP_123275170.1_Chryseobacterium  -------------------------SINSDSVNLLEPMF-VSNSSNETLILVPNSAGEPI 34
                                                                   :  :      :.  .: : *:.* *:

AQX14441.1_Agrobacterium         SFLDLGKAIGRKTEHVAFHAVKLPRIGTTDDAGFREEITRLTNKVAEELSLNANLPTIIY 99
SulM_TE                          NFVDMSRAMPEQASDVAMFGVKLPRTEVDSDGAMLEEVRRLSNAVCDDLLAATDLPAIVF 99
WP_081284758.1_Chromobacterium   SFIEMGRAFSAAGVPLAACAVNLPRNEVDDDAAMVAEVERLAAEVCDELGRMSSLPLMVF 89
WP_088511513.1_Pseudomonas       SFIETGRELAARNGRLAAYAVNLPRNEIDDDAMMVGEVERLTAEVCDSLAQETALPIVVF 92
WP_056247584.1_Flavobacterium    SFSKTVEFIEKDSTNINFYGLKLPRTDPEEGQDMLDMLYKLSEDVVREIQEKVHTPIIIF 120
WP_123275170.1_Chryseobacterium  NFRDLTNSIEAISKEINIYCVKFPRTPLKENENHEKKRKLLLKDLVAEVKEKIRGSITIY 94
                                 .* .  . :      :    :::**    ..         *   :  .:         ::

AQX14441.1_Agrobacterium         GQCNGSALAISLARHLAQETRDVAGLFIGGALMRT--SLSPADNRSDEEILSFLTSLGST 157
SulM_TE                          AQCNGSALALAITRELVRRSADVRALCIGGALMRT--VTGKRDTRTDDEILAFLGKAGST 157
WP_081284758.1_Chromobacterium   AQCNGSALALGVVREWKRRGADLRALCIGGALLRT--EPTPKDVRSDEQIVAFLGSIGST 147
WP_088511513.1_Pseudomonas       AQCNGSALAISIARELTRRGADLRALCIGGALLRT--EHTRKDTRSDETIVDFLRSLGAT 150
WP_056247584.1_Flavobacterium    GQCNGNVLAISIAEILQRINFNVEAICIGAIFPRTRKSLG-FGKRNNSEVVKFLTSLGST 179
WP_123275170.1_Chryseobacterium  GQCNGTALAIGLADELRNNDINIECLYLGALFPLEMKNLVENDKRTDDHILNMLGELGGV 154
                                 .****..**:.:.    .   ::  : :*. :          . *.:. :: :* . *..

AQX14441.1_Agrobacterium         IPSDPAEAAFFLHDFRYDCGFANAYYNQLIE-EVRGHTLEPLNIPLTCMVGTNDEMVRGY 216
SulM_TE                          LPAQPDEQAFFLHDFRYDGWLADVYYNHLVD-LMSRGALEVVDIPVWCLVGSEDPLVPNY 216
WP_081284758.1_Chromobacterium   LPAEADERAFFLHDFSYDCHMADSYYNHLVR-ERAAEAGERIGAPVFCLVGTEDPIVSGY 206
WP_088511513.1_Pseudomonas       LPTRPDEFEFFMQDFRYDCSMADAYYNHLLA-EIDGGRVARIGAPIFNLVGTLDAIVPNY 209
WP_056247584.1_Flavobacterium    IPTDPLDQEFFIKNFRYDSDLAVAGFNHYLEATKKRNRFDKFRMPLHFITGSIDPITKGY 239
WP_123275170.1_Chryseobacterium  FPTESPYKEFFLENIRYDSMMAQTGFHYFYH-QIKEKKFKKFDFPIHSVVGSEDTVTKYY 213
                                 :*:      **:.:: **  :*   ::              .  *:  :.*: * :.  *

AQX14441.1_Agrobacterium         QTDYRLWGKISQEIRLVEYHGFGHYLLRDCPELIADTMLEACSNFCHLESLAS-----   269
SulM_TE                          PVRFQDWSHIGRPVQLVEYAGIGHYLLRDCPEAIARAVGSVWEHVSCKGVTA------   268
WP_081284758.1_Chromobacterium   AERYRDWGLLSDEVSLIEYPGIGHYLLRDCPAELAASLADIWRRVGGK----------   254
WP_088511513.1_Pseudomonas       RARHRDWTHLSDDVQLVEYPGVGHYLLRDCPDLVADTLCSVWKDVRNEG---------   258
WP_056247584.1_Flavobacterium    KRKHKRWHLFAETVDISVIENHGHYLLRDAPAELGNILVNITNKNRKTVNIEYENENK   297
WP_123275170.1_Chryseobacterium  SFRYRKLKKYSNKVSLHVIKGVGHYLHRDAYTQLANILIKMRKK--------------   257
                                    .:     .  : :    . **** **.   :.  : .
```

Supplemental Figure S11. Sequence alignment with SulTE domain homologous proteins. SulTE protein sequence was used as query for protein BLAST and sequences showing less than 70% sequence similarity were selected. Further screening was done by using structural features of SulTE domain. Sequences were selected by identifying presence of 1) "QCN" residues (highlighted yellow) at the active site, 2) two out of three Arginine residues (highlighted blue) for interacting with substrate residues and 3) catalytic residue Aspartate on position II (highlighted red).

SulM protein from *Paraburkholderia acidicola*

NCBI Reference Sequence: AOZ21320.1

<u>SulM Thioesterase Construct</u>

```
                          2723
      GSSHHHHHHS SGLVPRGSHM ASEESSSIVL MAGDPATAKA VVVCVANAAG GPVNFVDMSR
2758  AMPEQASDVA MFGVKLPRTE VDSDGAMLEE VRRLSNAVCD DLLAATDLPA IVFAQCNGSA
2818  LALAITRELV RRSADVRALC IGGALMRTVT GKRDTRTDDE ILAFLGKAGS TLPAQPDEQA
2878  FFLHDFRYDG WLADVYYNHL VDLMSRGALE VVDIPVWCLV GSEDPLVPNY PVRFQDWSHI
2938  GRPVQLVEYA GIGHYLLRDC PEAIARAVGS VWEHVSCKGV TA*
```

<u>SulM PCP-Thioesterase Construct</u>

```
                          2660
      GSSHHHHHHS SGLVPRGSHM ERLTEIFRGV LGHAAFGIRD DFFDLGGDSF KAIRIAAKYG
2700  PPLEVTDIYD HPTIEALAEH LEHASEESSS IVLMAGDPAT AKAVVVCVAN AAGGPVNFVD
2760  MSRAMPEQAS DVAMFGVKLP RTEVDSDGAM LEEVRRLSNA VCDDLLAATD LPAIVFAQAN
2820  GSALALAITR ELVRRSADVR ALCIGGALMR TVTGKRDTRT DDEILAFLGK AGSTLPAQPD
2880  EQAFFLHDFR YDGWLADVYY NHLVDLMSRG ALEVVDIPVW CLVGSEDPLV PNYPVRFQDW
2940  SHIGRPVQLV EYAGIGHYLL RDCPEAIARA VGSVWEHVSC KGVTA*
```

Supplemental Figure S12. Protein sequences of SulM thioesterase and PCP-thioesterase constructs. The twenty residues in red represent the N-terminal His_6 tag and thrombin cleavage site, which remained in place for the crystallization experiment. The thioesterase domain begins at residue Ser2728. The thioesterase domain construct begins at Ala2723, containing several residues from the linker following C-terminal α-helix of the PCP; Ala2984 is the natural C-terminus of the SulM protein. The catalytic triad is highlighted in yellow. The PCP-Thioesterase construct constrains the PCP domain (blue), Glu2660 to Ala2723, followed by the thioesterase domain. The GGDS pantetheine binding motif of the PCP is underlined in blue. The catalytic triad is highlighted in yellow, with the mutation cysteine to alanine highlighted in cyan.

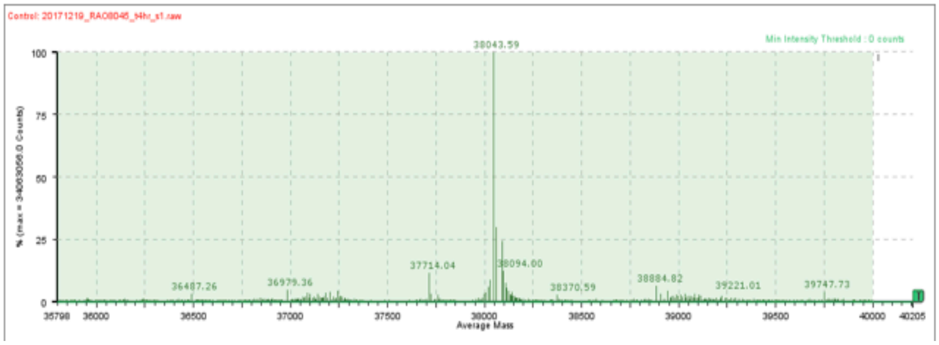

Supplementary Figure S13. Loading of sulfazecin tripeptide mimic to PCP3-TE C2818A. Deconvoluted HRMS spectrum confirmed successful stable loading of tripeptide mimic to the PCP_3-TE* C2818A didomain. Calc. 38042 Da (-Met), found 38043 Da.

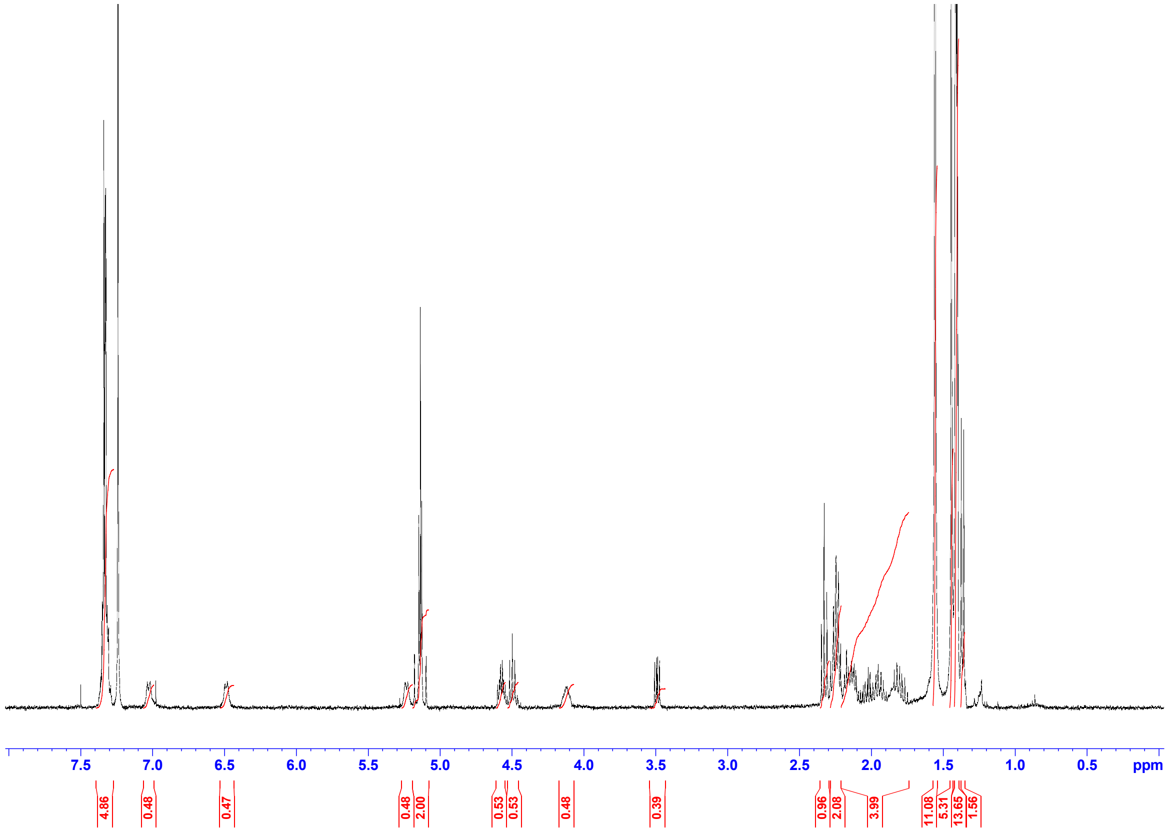

Supplementary Figure S14. ^{1}HNMR spectrum of Compound 3.

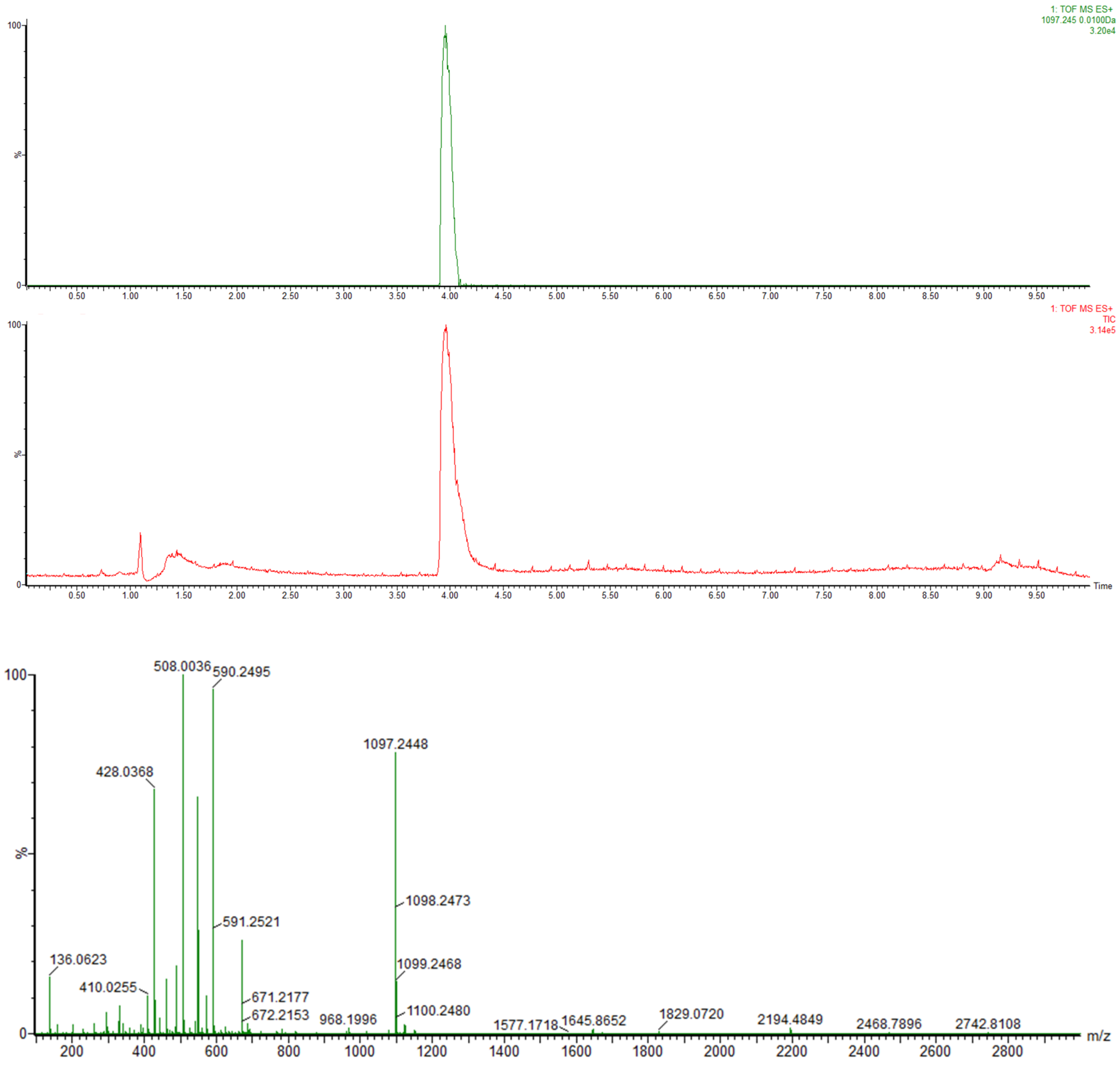

Supplementary Figure S15. Mass spectrometry of tripeptide-CoA thioester. Extracted ion chromatogram and total ion chromatogram of **4**. Positive mode ESI: *m/z* = 1097. 245 ± 0.01. Lower panel shows ESI (+) mass spectrum of **4**.

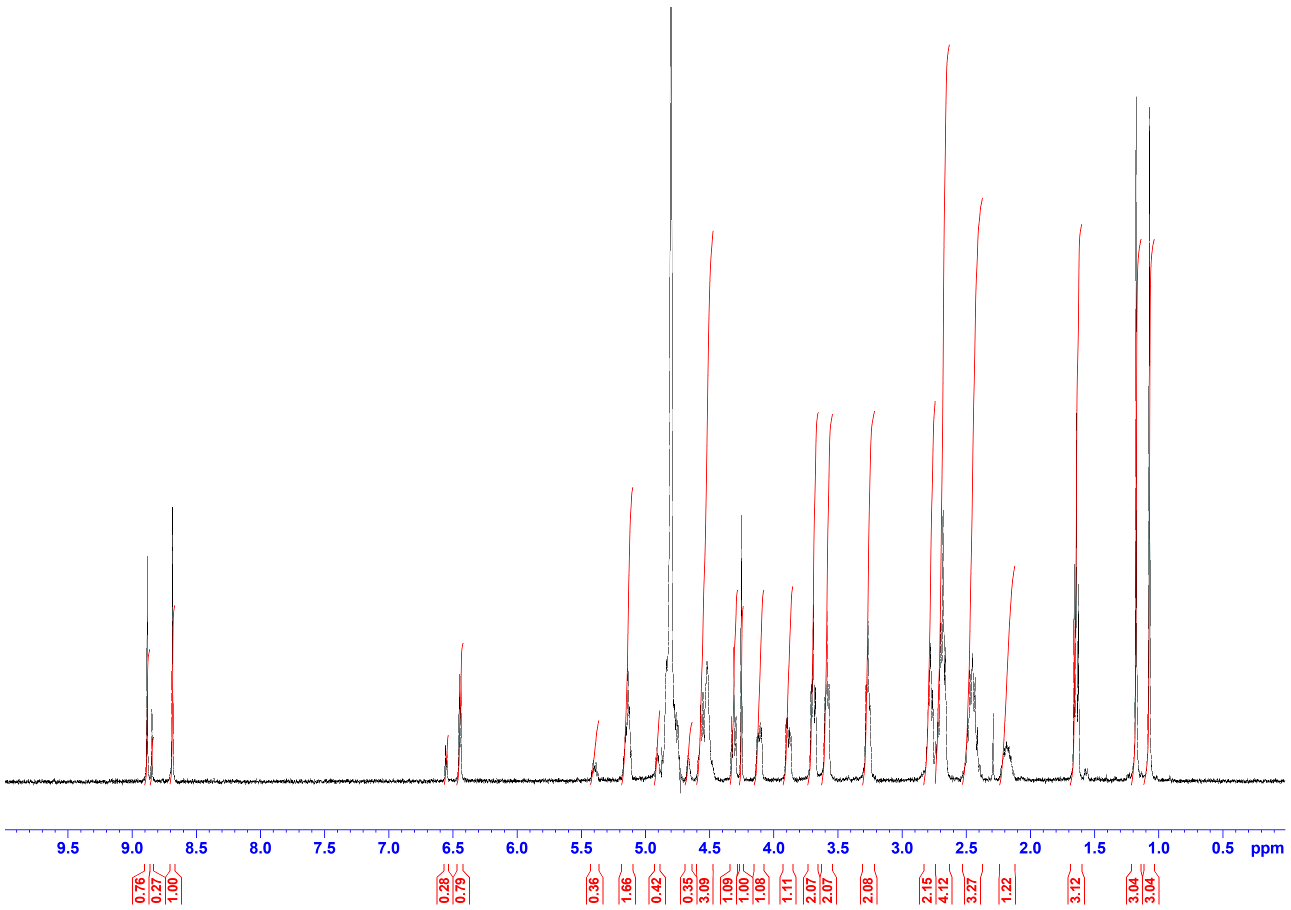

Supplementary Figure S16. 1HNMR spectrum of Compound 4.

Supplemental Tables

Table S1. Secondary structure of lid regions of structurally characterized NRPS thioesterase domains.

Protein	PDB	β6-β7 Loop	# AA	β7-β8 loop details	# AA
SulM	**8W2C**	Gly2844 – Val2918	75	Gly2923 – Val2946	24
		αL1 Asp2860 – Ala2870	11	α Tyr2932 – Ile2942	11
		αL2 Pro2878 – Ser2907	30		
SrfA-C	**2VSQ**	Val1146 – Asp1211	66	Thr1216 – Arg 1236	21
	**1JMK**	αL1a Thr1161 – Asn1171	11	No α helices.	
		αL1b Glu1177 – Ser1181	17		
		αL2 Lys1185 – Asn1201			
Vlm2	**6ECE**	Leu2489 – Ile2589	101	Thr2594 – 2615	22
		αL1a Thr2506 – Met2517	12	α Ala2598 – Ala2612	15
		αL1b Tyr2535 – Asn2548	14		
		αL1c Pro2550 – Phe2555	6		
		αL2 Glu2558 – Met2573	16		
EntF	**5JA1**	Leu1164 – Phe1232	69	Ala1241 – Ile1259	19
		αL1a Pro1169 – Trp1174	6	α Ser1250 – Ser1256	7
		αL1b Glu1185 – Ala1200	16		
		αL2 Thr1207 – Thr1225	19		
NocB	**6OJD**	Val1805 – Val1865	61	Val1869 – Leu1892	24
		αL1a Asn1809 – Ala1813	5	α Asp1877 – Arg1882	6
		αL1b Asp1818 – Leu1830	13		
		αL2 Ser1836 – Tyr1856	21		
ObiF	**6N8E**	Gly1172 – Leu1246	75	Gly1251 – Val1274	24
		αL1a Asp1179 – Gly1188	10	α Asp1259 – Ser1271	12
		αL1b Ser1191 – Ile1202	12		
		αL2 Asp1210 – Glu1235	26		
FenB	**2CB9**	Val1149 – Asn1202	54	Glu1206 – Tyr1231	26
		αL2 Pro1172 – Leu1193	22	α Ser1215 – Leu1220	6
				α Trp1223 – Ala1226	4
SkyXY	**7CRN**	Leu3753 – Val3844	92	Ala3849 – Ile3875	24
		αL1a Ala3764 – Gln3758	5	α Pro3856 – Val3871	16
		αL1b Asp3770 – Val3782	13		
		αL1c Asp3795 – Arg3806	12		
		αL2 Asp3814 – Phe3832	19		
AB3403	**4ZXI**	Ile1161 – Ile1260	100	Ala1265 – Leu1286	22
		αL1a Ile1167 – Val1171	6	α Glu1277– Leu1281	5
		αL1b Asp1176 – Gly1193	18		
		αL1c Pro1199 – Glu1205	7		
		αL1d Ser1207 – Ala1221	15		
		αL2 Asp1230 – Thr1250	21		

Table S2: Crystallographic Diffraction and Refinement Data.

**Data collection**	SulM Thioesterase	SulM PCP-Thioesterase
**NCBI Accession Code**	WP_096724622.1	WP_096724622.1
**Residues**	2778 – 2984	2660 – 2940
**PDB**	**8W2C**	**8W2D**
**Beamline**	SSRL BL 9-2	SSRL BL 9-2
**Wavelength (Å)**	0.979 Å	0.979 Å
**Resolution range (Å)**[a]	50.0 – 1.9 (2.0 – 1.9)	47.8 – 2.7 (2.84 – 2.70)
**Space group**	$P\ 2_1$	$P\ 2_1$
**a, b, c (Å)**	47.09, 83.20, 69.36	60.1, 79.1, 70.5
**α, β, γ (°)**	90.0, 101.4, 90.0	90.0, 93.5, 90.0
**Total reflections**	276587	72068
**Unique reflections**	39586	17876
**Multiplicity**	7.0 (7.0)	4.0 (3.9)
**Completeness (%)**	95.8 (95.8)	97.7 (94.5)
**Mean I/sigma(I)**	11.4 (1.5)	8.1 (1.4)
**R_{merge} (%)**	16.5 (223.9)	13.9 (94.9)
**R_{pim} (%)**	6.7 (90.3)	7.7 (53.2)
**$CC_{1/2}$**	0.99 (0.56)	0.99 (0.65)
**Refinement**		
**Resolution range (Å)**	42.26 – 1.90 (2.00 – 1.95)	40.46 – 2.70 (2.77 – 2.70)
**Reflections, refinement**	39525	17786
**Reflections, R_{free}**	1995	1768
**R_{work} (%)**	17.47 (31.08)	22.08 (28.55)
**R_{free} (%)**	21.49 (38.77)	25.69 (33.74)
**Protein residues**	507	642
**Ligands of interest**	--	2 (Pantetheine)
**Water molecules**	320	28
**RMS (bonds) (Å)**	0.009	0.003
**RMS (angles) (°)**	0.915	0.801
**Ramachandran favored (%)**	98.81	97.00
**Ramachandran allowed (%)**	1.19	3.00
**Ramachandran outliers (%)**	0.0	0.0
**Rotamer outliers (%)**	0.25	0.0

[a]Values in parentheses are for high resolution shell

Table S3. Potential uncharacterized β-lactam producing BGCs. Three BGCs identified from anti-SMASH that may produce uncharacterized β-lactam products. The Stachelhaus code residues in the adenylation domain of the terminal module with high similarity to the sulfazecin cluster are also included.

Cluster / bacteria	Anti-SMASH predicted product	Stachelhaus code*
Sulfazecin / Paraburkholderia acidicola	γ-Glu-Ala-DAP	DVWEMNADDK
Flavobacterium sp. Leaf82<br>WP_056247584.1<br>NZ_LMMA01000010.1	Orn-Gly-Asp-Asp-Xxx-Thr-DAP	DIWEINTDDK
Agrobacterium tumefaciens<br>BGC0001671<br>KY452017.1	Xxx-DAP	DIWEITADDK
Chryseobacterium sp. G0240<br>WP_123275170.1<br>NZ_RJTV01000006.1	Asp-Leu-D-Orn-DAP	DIWEVNTDDK

* Stachelhaus code residues in adenylation domain of the last modules
